# Comprehensive Characterization and Relative Quantification of α-Amylase/Trypsin Inhibitors from Wheat Cultivars by Targeted HPLC-MS/MS

**DOI:** 10.3390/foods9101448

**Published:** 2020-10-13

**Authors:** Sorel Tchewonpi Sagu, Lynn Zimmermann, Eva Landgräber, Thomas Homann, Gerd Huschek, Haydar Özpinar, Florian J. Schweigert, Harshadrai M. Rawel

**Affiliations:** 1 Institute of Nutritional Science, University of Potsdam, Arthur-Scheunert-Allee 114-116, 14558 Nuthetal, Potsdam, Germany; sorelsagu@uni-potsdam.de (S.T.S.); lyzimmer@uni-potsdam.de (L.Z.); e.landgraeber@live.de (E.L.); homann@uni-potsdam.de (T.H.); fjschwei@uni-potsdam.de (F.J.S.); 2 IGV-Institut für Getreideverarbeitung GmbH, Arthur-Scheunert-Allee 40/41, 14558 Nuthetal, Germany; gerd.huschek@igv-gmbh.de; 3 Department of Gastronomy, Faculty of Applied Science, University of Altınbaş, Büyükdere Cad. No 147, 34394 Esentepe-İstanbul, Turkey; haydarozpinar@gmail.com

**Keywords:** α-amylase/trypsin inhibitors, wheat cultivars, SDS-PAGE, peptides markers, relative quantification, mass spectrometry, LC-MRM-MS

## Abstract

The α-amylase/trypsin inhibitors (ATIs) are discussed as being responsible for non-celiac wheat sensitivity (NCWS), besides being known as allergenic components for baker’s asthma. Different approaches for characterization and quantification including proteomics-based methods for wheat ATIs have been documented. In these studies generally the major ATIs have been addressed. The challenge of current study was then to develop a more comprehensive workflow encompassing all reviewed wheat-ATI entries in UniProt database. To substantially test proof of concept, 46 German and Turkish wheat samples were used. Two extractions systems based on chloroform/methanol mixture (CM) and under buffered denaturing conditions were evaluated. Three aspects were optimized, tryptic digestion, chromatographic separation, and targeted tandem mass spectrometric analysis (HPLC-MS/MS). Preliminary characterization with sodium dodecyl sulfate polyacrylamide gel electrophoresis (SDS-PAGE) documented the purity of the extracted ATIs with CM mixture and the amylase (60–80%)/trypsin (10–20%) inhibition demonstrated the bifunctional activity of ATIs. Thirteen (individual/common) biomarkers were established. Major ATIs (7–34%) were differently represented in samples. Finally, to our knowledge, the proposed HPLC-MS/MS method allowed for the first time so far the analysis of all 14 reviewed wheat ATI entries reported.

## 1. Introduction

Wheat contains about 8% to 15% of proteins divided into the four so-called Osborne fractions according to their solubility: albumins or water soluble fraction, globulins soluble in diluted saline solution, gliadins extracted with 70 percent ethanol, and glutenins (partly soluble under mild acidic or alkaline conditions) [1]. Gliadins and glutenins considered as the gluten fraction represent 80% to 90% of the total wheat proteins. The α-amylase/trypsin inhibitors (ATIs) appear to be the major group of non-gluten fractions consisting of albumins and globulins [2]. ATIs are bifunctional proteins with the ability to inhibit both amylase and proteases activities, describing their role in cereals [3,4]. However, it should be taken into account that there are differences in the enzyme inhibitory activity of ATIs in cereals, mammals, and insects; ATIs have a stronger effect on insect α-amylases than mammals or cereal enzymes [5]. ATIs are located in wheat endosperm, where they are also involved in carbohydrate metabolism due to their ability to regulate the glycolytic activity of amylase [6]. α-amylase inhibitors are relatively small proteins (12 to 16 kDa) containing 124 to 168 amino acids with 10 cysteine residues, allowing the formation of 5 intra-molecular disulfide bridges; with the exception of the inhibitor α-amylase 0.53, whose structure includes nine cysteines and four disulfide bridges [7,8]. This results in the generation of a compact three-dimensional structure capable of withstanding digestion and thermal processing, and is characterized by the formation of α helices and β leaflet structural elements [9,10,11]. Their primary structures are often similar but do differ slightly, exhibiting a highly conserved secondary structure [3]. The disulfide bridges are presumably involved in the bifunctional structural ability of ATIs to inhibit both amylase and proteases activities while resisting high processing temperatures [12].

The family of wheat ATIs includes the 12 kDa monomeric (α-amylase inhibitor 0.28), the 24 kDa homodimeric (α-amylase inhibitor 0.19 and 0.53, WDAI-3) and the 60 kDa heterotetrameric forms, which are often referred to as chloroform/methanol mixture (CM) proteins because of their solubility in chloroform/methanol mixtures [7,13,14]. Heterotetramers generally consist of a CM1 or CM2 subunit in combination with a CM16 or CM17 subunit and two CM3 subunits and their inhibitory activity is related to the combination of the subunits [15]. In wheat, the relative distribution of ATIs is as follows: 50% heterotetramers (CM proteins), 33% homodimers (0.19), and 17% monomers (0.28) [3,7]. Compared to the gluten fraction, ATIs contain a higher proportion of essential amino acids, such as tryptophan, lysine, and leucine, which has a higher nutritive value for the human nutrition [2]. However, ATIs have been recently discussed as being responsible for select types of wheat disorders, the so called non-celiac wheat sensitivity (including as allergens for baker’s asthma) [16]. They stimulate the innate immune response in the intestine, which in turn leads to an adaptive immune response, in that the myeloid cells activated in the intestine probably migrate into the peripheral tissue [17,18]. ATIs can also promote the development of insulin resistance and inflammation in adipose tissue [18]. Wheat monomeric ATIs as well as subunits of the wheat dimeric α-amylase inhibitor and the tetrameric α-amylase inhibitor have been reported as allergens [19,20,21]. Junker et al. [22] showed that ATIs are strong inducers of innate immune responses in humans, activating the Toll-like receptor-Myeloid Differentiation factor 2-cluster of differentiation 14 complex (TLR4–MD2–CD14) and eliciting strong innate immune effects [22]. They showed that members of the wheat ATIs family promote the release of inflammatory cytokines in vitro by activating monocytes, macrophages, and also dendritic cells. An in vivo study with C57BL/6 mice conducted by Zevallos et al. [17] indicated that in the ATI-administered group, the number and activation of myeloid cells in the mesenteric lymph nodes was significantly increased compared to the control group.

As with other allergens, the allergenic effect of ATIs is dose-dependent and related to their quantity present in different types of wheat consumed. Thus, if the ELISA method has often been used to quantify differences in IgE-binding proteins [23], research has recently led to the development of new proteomics-based methods applying instrumental analysis, such as chromatography combined with tandem mass spectrometry (HPLC-MS/MS). Prandi et al. [24] accomplished the analysis of the allergen Tri a 30 content (the CM3 α-amylase/trypsin inhibitor) by using an ultra-performance liquid chromatography-mass spectrometry (UPLC-MS/MS) system. They identified the quantifier marker peptides of the protein CM3 from the salt soluble extract. Rogniaux et al. [13] also used salt-soluble fraction to develop a targeted MS/MS methods to detect and quantify relative abundance of some selected wheat allergens from three different wheat species. They were able to detect five ATIs (AAI 0.28, AAI 0.19, CM1, CM2, and CM3). Zevallos et al. [17] characterized the biologic activity of ATIs from 38 different gluten-containing and gluten-free unprocessed and processed products. Here, ATIs were extracted successively three times with ammonium bicarbonate buffer followed by an ammonium sulfate fractionation. After tryptic digestion, resulting peptides were used for nanoscale UPLC-MS analysis. In addition, the same extraction procedure was used by Geisslitz et al. [25] to quantify six predominant ATIs (0.19, 0.53, 0.28, CM2, CM3, and CM16) from eight different cereal cultivars. In this work, tryptic digestion was performed in order to produce peptides before targeted LC−MS/MS analysis and untargeted based absolute quantification algorithm (iBAQ). More recently, 15 commercial wheat cultivars were used to analyze the ATI content [26]. The authors subsequently tried to maximize extraction of ATIs using isopropyl alcohol/dithiothreitol buffer (50 °C for 30 min) and then 8 M urea extraction buffer (at room temperature for 45 min). Supernatants from both extracts were pooled before performing the tryptic digestion. The analysis was finally carried out using a liquid chromatography-multiple reaction monitoring-mass spectrometry (LC-MRM-MS) [26].

From the foregoing examples, it emerges that salt solutions (either sodium chloride or precipitation by ammonium sulfate) have been generally used to extract ATIs from wheat samples prior to their digestion and analysis. Moreover, it appears that in the studies mentioned only some of the ATI constituents contained in the wheat were analyzed. It therefore emerges that the development of proteomics-based analytical methods for the characterization and quantification of ATIs, both regarding the diversity range of wheat cultivars analyzed as well as the coverage of all the wheat ATIs currently reported in the online protein database, remains a topical issue. To date, there are 22 entries referring to the wheat ATIs in the online UniProtKB database, of which 14 are reviewed. In this regard, our challenge in this work is then to propose an alternative proteomics-based method for the characterization and relative quantification of all the reviewed wheat-ATI entries using a wide variety of samples. In total, 25 German and 21 Turkish wheat cultivars samples are analyzed. The aim is to characterize in the first instance those ATIs extracted by using the chloroform/methanol mixture, followed by the salting out and thereafter try to get the highest possible yield using denaturing conditions. A targeted multiple reactions monitoring (MRM) high-resolution tandem mass spectrometric based-method needs to be developed and statistically proofed to analyze the tryptic digested ATIs. The alternative extraction methods using 100 mM ammonium bicarbonate buffer (containing 4 M urea) are checked to maximize the ATIs extraction, and thus improve their relative distribution and quantification in wheat samples.

## 2. Materials and Methods

### 2.1. Materials

#### 2.1.1. Samples Collected from Germany

Twenty-five wheat samples of different cultivars of the genus *Triticum* were selected for analysis and characterization, including 10 whole flours and 14 flour/grain fractions (whole-meal flour, centrifuged flour, passage flour, grain bran, and sling bran fractions of cultivars *Julius* 2017, *Ponticus* 2017, and *Elixer* 2018) belonging to *Triticum aestivum* L. The last cultivar was an old wheat cultivar *Ackermanns Bayernkönig*, a crossing between wheat and spelt wheat. The wheat cultivars came from different locations of the Brandenburg Region in Germany. The harvest years were between 2017 and 2018. The samples were in part kindly provided by VERN e.V, Raben, Germany and the Institut für Getreideverarbeitung GmbH, Nuthetal, Germany. For the production of the flour and grain fractions, the Buhler automatic milling machine and the Buhler bran centrifuge were used according to the “Standard Methods for Grain, Flour and Bread” [27]. A schematic representation and short description of the milling process are shown in Supplementary Figure S1. Detailed information on the wheat samples is summarized in the Table 1. Further, two commercially available samples—whole-meal (Biokorn GmbH & Co. KG, Aalen, Germany) and extracted wheat flour type 405 (Kunstmühle Reisgang, Pfaffenhofen, Germany)—were also analyzed.

#### 2.1.2. Samples Collected from Turkey

Samples were collected from selected regions of Turkey with different climates and from harvests of different years. Table 2 documents some characteristics of these samples. The sample numbers were randomized in Germany and are given in the column “Sample”.

#### 2.1.3. Other Chemicals

A synthesized peptide with the sequence GWGG (Bachem AG, Bubendorf, Switzerland) with an *m/z* ratio of 376.2 was used as internal standard (IS) to check the performance of the MS analysis. IS stock solution of 0.1 µg/mL was prepared and then mixed with samples before the LC-MS/MS analysis for a final concentration of 0.01 µg/mL. Using an in silico digestion by Skyline Software (MacCoss Lab Software, University of Washington; https://skyline.gs.washington.edu), the formation of this peptide by the tryptic digestion of the wheat samples investigated was checked and excluded [28]. Whey protein (Biopure, lot JE 002-8-415) was obtained from Davisco Foods International, Inc. (Le Sueur, MN, USA). The powder contained 98% protein, as determined by Kjeldahl analysis (N × 6.38), 0.1% fat, 1.9% ash, and 4.7% moisture, as specified by the supplier. The major whey protein fraction was ca. 93.4% β-lactoglobulin, as determined by sodium dodecyl sulfate polyacrylamide gel electrophoresis (SDS-PAGE) [29]. This protein is termed β-Lg and was used to monitor the tryptic digestion. Proteomics-grade trypsin (Sigma Aldrich, Steinheim, Germany) was used to perform the digestion of the proteins. The solvents used for the HPLC and the LC-MS/MS were of LC-MS grade and all the other chemicals were of analytical grade.

### 2.2. Methods

**Figure 1:**
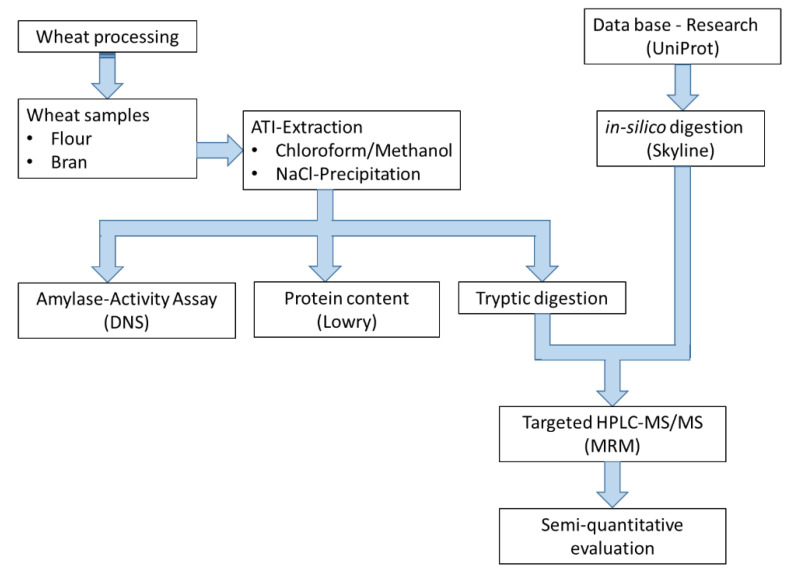
Workflow and methods applied for the analysis of α-amylase/trypsin Inhibitors (ATIs). Extraction was based on our recently published study [3]. DNS: α-amylase activity using a colorimetric method with 3,5-dinitrosalicylic acid reagent; Lowry: Assay for determining the protein content in in the extracted solutions; MRM: Multiple reaction monitoring

The following workflow (Figure 1) was used to develop a comprehensive method for the extraction, characterization, and identification of selected ATIs in wheat. The methods listed are described in detail in the following sections.

#### 2.2.1. Extraction of the ATIs Using the Chloroform/Methanol Method

The extraction of ATIs was carried out as reported in our previous study [3] using a chloroform/methanol (C/M) mixture in a ratio of 2:1. A short summary of the individual processing steps with their parameters is given in Supplementary Figure S2.

#### 2.2.2. Protein Determination

The protein content of the final extracted proteins was determined while applying the Lowry method [30]. The samples were diluted 1:4 with distilled water before analysis and an external calibration with bovine serum albumin (Fluka Chemie AG, Buchs, Switzerland) was applied.

#### 2.2.3. Inhibition of Amylase Activity

A modified 2,3 dinitrosalicylic acid reagent (DNS) method was used to determine the inhibition activity [31]. Briefly, 70 μL of the extract that contained ATIs was pre-incubated with 10 μL of 1 mg/mL porcine α-amylase (with a specific activity of 23 units/mg solid; Sigma Aldrich, Steinheim, Germany) and 120 μL of buffer (0.02 M sodium phosphate buffer, pH 6.9 with 0.006 M sodium chloride) for ten minutes at 37 °C. Subsequently, 200 μL of 1% soluble starch (Serva, Heidelberg, Germany) was added and incubated for another ten minutes at 37 °C. The reaction was then stopped and the colorimetric reaction was performed by adding 400 μL DNS (Alfa Aesar GmbH, Karlsruhe, Germany) and incubating for five minutes at 95 °C. After cooling to room temperature, 700 μL of distillated water was added and the absorption was measured photometrically at 540 nm (Hanna instruments, Vöhringen, Germany). The maltose produced can be quantified by external calibration ranging from 0.25 to 5 µmol maltose/mL (Sigma Aldrich, Steinheim, Germany). A negative test was prepared by mixing 70 µL of ATIs with 130 µL of sodium phosphate buffer. In addition to the extracts, positive controls with 50 µM and 1 mM acarbose solutions (Sigma Aldrich, Steinheim, Germany) were also performed. Residual activity was calculated and inhibition of amylase activity (IAA) expressed in percentage was evaluated as described in [3].

#### 2.2.4. Inhibition of Trypsin Activity

Modified azocasein protease assay, as described in our previous works, was used to test the trypsin inhibition activity of the extracted ATIs [31]. Briefly, 75 µL of extracted ATIs was mixed with 30 µL of 0.5% sodium bicarbonate buffer pH 8.3 and 20 µL of 10 mg/mL trypsin solution (Sigma Aldrich,). For the control, 20 µL of trypsin was mixed with 105 µL of buffer. 125 µL of 2.5% azocasein solution (Sigma-Aldrich Chemie GmbH, Steinheim, Germany) was added and the mixture was incubated at 37 °C for 30 min. Then, 100 µL of the mixture was mixed with 400 µL of 5% trichloroacetic acid solution, incubated at room temperature for 5 min, and centrifuged for 5 min at 10,000× *g*. A total of 400 µL of the above solution was mixed with 1200 µL of 500 mM NaOH solution and the absorbance was taken at 440 nm. The trypsin activity was expressed as the amount of activity that gave a change of one unit of absorbance at 440 nm and the inhibition of trypsin activity was calculated as reported in [3]. The results were expressed as percentage of inhibition.

#### 2.2.5. SDS-Page

The C/M extracts containing ATIs were analyzed using sodium dodecyl sulfate-polyacrylamide gel electrophoresis (SDS-PAGE). The experiments were carried out under reducing and denaturing conditions, as described formerly [3]. Briefly, the ATI extracts were mixed in equal parts with sample buffer (pH 8.4 containing glycerol, 2-mercaptoethanol, SDS and Coomassie blue G250) and denatured for five minutes at 95 °C. Twenty-five µL of the mixture and 5 µL of the broad range PageRuler Plus marker (Thermo Fisher Scientific, Carlsbad, CA, USA) were applied to a 12% Bis-Tris gel (Thermo Fisher Scientific, Carlsbad, CA, USA) and separated at 50 mA per gel. The gels were stained using Coomassie Brilliant Blue R-250 solution overnight at room temperature. The gels were destained with 10% acetic acid and then scanned using the Bio-500 Professional VIS Gel Scanner (SERVA Electrophoresis GmbH, Heidelberg, Germany). The band intensity and protein composition were evaluated with the ImageLab software (Bio-Rad Laboratories Ltd., Hemel Hempstead, UK).

#### 2.2.6. In-Solution Tryptic Digestion

While using trypsin, a specific digestion after the amino acids lysine and arginine is possible. For this purpose, 50 μL of the extract was mixed with 390 μL of 50 mM digestion buffer (50 mM ammonium bicarbonate solution; Carl Roth GmbH, Karlsruhe, Germany) and the proteins reduced by using 5 μL of 45 mM Tris(2-carboxymethyl)phosphine (TCEP; Carl Roth GmbH, Karlsruhe, Germany) for 15 min at 50 °C. Thereafter, 5 μL of 100 mM iodoacetamide (Sigma Aldrich, St. Louis, MO, USA) was added to the reaction mixture and incubated for a further 15 min at room temperature in the dark. Accordingly, the cysteine residues were alkylated and a renewed formation of disulfide bridges was prevented. The proteins thus treated were digested with 50 μL of activated trypsin (10 µg/mL; Sigma Aldrich, Steinheim, Deutschland) and incubated for three days at 37 °C under the shaking conditions. The final reaction volume was 500 μL with an app. enzyme:substrate ratio of 1:50. The reaction was stopped by the addition of 25 µL of 10% trifluoroacetic acid (TFA). We assumed that the low tryptic inhibition activity measured for the ATIs extracted would be further diminished by the reduction and alkylation steps (loss of secondary/tertiary structure—further denaturation), and thereafter their digestion by trypsin (which was then present in surplus) would be effective. A negative control (buffered blank) was carried out along with the samples. It contained the same amount of distilled water instead of the extract and the samples were then analyzed.

#### 2.2.7. Development of a Multiple Reaction Monitoring (MRM) Assay Using HPLC-MS/MS

**Figure 2:**
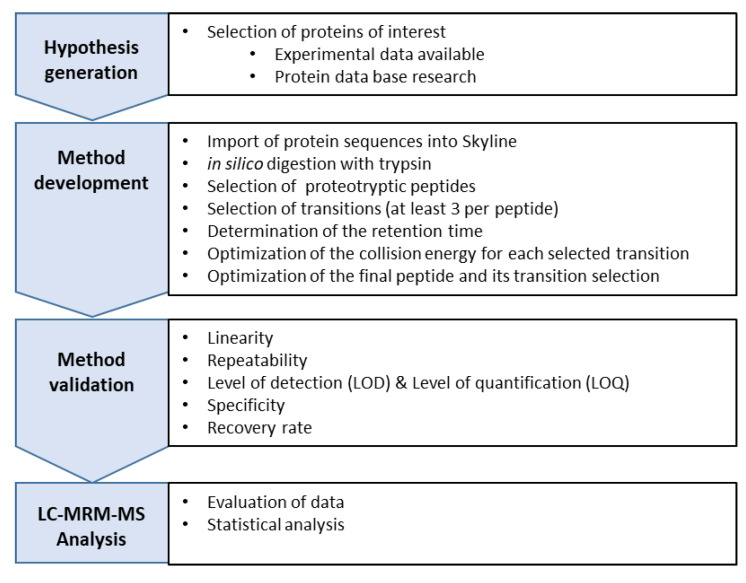
Workflow for developing a Multiple Reaction Monitoring (MRM) assay using a high performance liquid chromatography mass spectrometry HPLC-MS/MS.

The aim of the Multiple Reaction Monitoring (MRM) assay, while applying HPLC-MS/MS, was to develop a highly specific and sensitive method to detect the frequency of one or more known ATIs within a sample. For the method development, a defined workflow was followed and corresponding steps are outlined in Figure 2.

##### Hypothesis

The information about wheat ATIs was obtained from the UniProt online database (https://www.uniprot.org/) and while considering the published data [13,17,24,25]. A total of 14 reviewed records with information extracted from literature and curator-evaluated computational analysis was compiled from UniProt database. The compilation is supplied as Supplementary Table S1.

##### Method Development

The sequences of the 14 selected ATIs were imported into the MacCoss Lab Skyline software via a FASTA (fast-all) data file. FASTA is a program developed by Pearson et al. for database search and sequence comparison of proteins and DNA [32]. The Skyline program performs an in silico digestion with the imported sequences of the target proteins. Skyline generates a list of all possible peptides, taking into account the probability of their formation. Which peptides are best suited for the analysis is determined by the ionization capability, signal strength, and specificity of the peptides for a certain protein [33]. For this purpose, the following settings were considered: Trypsin was defined as the enzyme applied for peptide generation, partials equal to zero (this option allows skipping a number of cutting positions), the background proteome was set for wheat (it contained all the available protein entries as downloaded from the UniProt database), the length of the peptides was set to 5–25 amino acids and the modification considered was the carbamidomethylation referring to the alkylation of the cysteine residues by iodoacetamide. Two to four transitions with the highest possible signal strength were selected per peptide. Further general criteria used to select the peptides and transitions included a mas-to-charge ratio of two for the precursors, the transition charge was mostly set at one, and the analysis was performed in positive mode. The specificity of a potential peptide was tested using the BLAST algorithm (Basic Local Alignment Search Tool) of the UniProt database.

The optimization process was carried out for each and every peptide generated for every selected ATI (Supplementary Table S2). Thereafter, the following three steps were successively monitored: (1) determination of the retention time of the peptides, (2) optimization of the collision energy, and (3) determination of the signal strength of the selected transitions (peak area). An exemplary process of optimization was documented for ATI protein P17314 in Figure S3 (supplementary data). Different gradients and columns were tested for the optimal separation and the final operating conditions are summarized in the following.

Tryptic digested proteins were analyzed using an Agilent Infinity 1260 system with a binary pump, multicolumn thermostat, and auto sampler equipped with an Agilent G6470A Series Triple Quad mass spectrometer (both from Agilent Technologies Sales & Services GmbH & Co.KG, Waldbronn, Germany) coupled with an electrospray (ESI) source operating in positive ionization mode. Peptides were separated using a Kinetex C8 analytical column, (2.6 µm, 100 A, 150 × 4.60 mm; Phenomenex, Torrance, CA, USA) set at a temperature of 30 °C. The mobile phase was composed of eluent A (0.1% formic acid) and eluent B (100% acetonitrile), at a flow rate of 0.5 mL min^−1^. Following mobile phase gradient conditions were applied: 95% A from 0 to 2 min, 95–50% A from 2 to 18 min, 50–5% A from 18 to 19 min, 5% A from 19 to 22 min, 5–95% A from 22 to 23 min, and 95% A from 23 to 28 min. The column equilibration time between each run (post run) was 4 min at 95% A. A total of 10 µL of digested samples was injected. The desolvation gas temperature in the ionization source was set at 275 °C. The gas flow was 11 l/min, the nebulizer pressure was 35 psi, the fragmentor voltage was set at 130, and the dwell time was 20 ms. The collision gas used was nitrogen. Detection was performed in the multiple reaction monitoring (MRM) mode, in which a specific transition was monitored at a specific time according to the retention time of the peptides. The MS-Data collection was performed between 3 and 21 min, whereby before and after it the eluent was directed into waste. The relative abundance of each peptide was measured considering the total area of all the transitions analyzed.

##### Validation of the HPLC-MS/MS

To provide quality assurance and to show that the developed method of analysis was suitable for fulfilling the specific task of analyzing the ATIs, the following assignments were undertaken. The German wheat cultivar “*Julius*” (Table 1) was used to produce a protein extract rich in ATIs (Section 2.2.1). This extract was used in the validation experiments together with the internal peptide standard GWGG. Here, the approach of [13] was followed, since our intention was to semi-quantify the content of the ATIs by measuring their relative abundance and since no specific peptides were available to conduct absolute quantification. The synthesis of such peptides is possible, but due to financial limitations this was not realizable.

Different amounts of the CM extracted ATIs (50–2500 ng protein content) as well as internal standard (5–250 pg) were injected to determine the linearity of response for the selected quantifier peptides (Supplementary Table S3). Eight concentrations of each were measured in triplicate. To determine the repeatability, samples were repeatedly injected and the signal strength (peak area) was measured. The measurements were performed on different days to provide the inter-day behavior by calculating the standard deviation.

The internal standard was used to indicate the matrix effects. For this purpose, the internal standard dissolved in distilled water and in sample was injected and the response was measured. Mean values and standard deviations were calculated for each sample. The peak area of the internal standard in water was set to 100% and compared with the peak areas of that in the matrix.

##### Normalization

For the normalization, and to take into account the matrix effect, the ratio internal standard peak area in the matrix/internal standard peak area in water was determined. The resulting factor was applied to the peptide peak area. The normalized peptides’ peak areas were corrected to 1 mg wheat flour. Average values and standard deviation were then calculated.

#### 2.2.8. Extraction of the ATIs Using Ammonium Bicarbonate Buffer

To have a more comprehensive detection and quantification of ATIs, we also performed the extraction of wheat ATIs using 100 mM ammonium bicarbonate containing 4M urea (Ambi/urea buffer). To this end, three different procedure options were tested (Figure S4, see supplementary data). Two wheat samples with higher content of ATIs (according to the C/M method) served as test material for the optimization of the extraction method. The option with the highest ATI yield was then used for the extraction of all the other wheat samples. Finally, relative ATIs contents obtained with the C/M method and the Ambi/urea method were compared.

##### Ambi/Urea Extraction

In the first extraction option, flour samples were mixed with 0.925 mL of extraction buffer. A total of 25 µL of TCEP (0.25 M) and 25 µL of IAA (0.25 M) were added and samples (S11, with a low and S20 with a high ATI content according to the C/M method) were then shaken in the dark for one hour at 95 rpm. This was followed by centrifugation for ten minutes at 7000× *g* and 4 °C. A total of 0.4 mL of the supernatant was collected from all samples and transferred to a 1.5 mL microtube and mixed in sequence with 165 µL digestion buffer and 20 µL trypsin (4 mg/mL). The samples were then incubated overnight at 37 °C and 300 rpm. To stop the reaction, 15 µL of 40% formic acid (*v/v*) was added.

The second option consisted of adding 1 mL of the extraction buffer to the samples. After shaking at 95 rpm for one hour, samples were centrifuged at 7000× *g* and 4 °C for ten minutes. A total of 0.4 mL of the supernatant was then collected and transferred to a 1.5 mL microtube. To the supernatant (in the final method, 10 µL of 0.5 mg/mL β-lactoglobulin in 100 mM ammonium bicarbonate was used to monitor the tryptic digestion and was added at this stage of the workflow), 10 µL of TCEP (0.25 M) was added, followed by 20 min incubation at 50 °C. The samples were then mixed with 10 µL of IAA (0.25 M) and incubated for 20 min at 50 °C in the dark. Thereafter, 135 µL digestion buffer and 20 µL trypsin (4 mg/mL) were successively pipetted to the samples and the whole was incubated overnight under the conditions described above. A total of 15 µL of a 40% formic acid (*v*/*v*) was added the following day to stop the reaction.

A third option of Ambi/urea extraction was also performed. In this case, samples were mixed with 1 mL extraction buffer, shaken for one hour (95 rpm, room temperature), and then centrifuged (7000× *g*, 10 min, 4 °C) as in option 2. Subsequently, 0.4 mL of the supernatant was transferred to a new 1.5 mL microtube. A total of 10 µL of 0.25 M TCEP and 0.25 M IAA were added to the supernatant and incubated in the dark for 30 min at 50 °C and 300 rpm. Here, the reduction and the alkylation reactions were achieved at the same time. In a next step, 135 µL digestion buffer and 20 µL trypsin (4 mg/mL) were added and the subsequent experimental steps were similar to those of option 2.

All extracts obtained using the different protocol options were stored at −20 °C until further analysis. Prior to the mass spectrometric analysis, the samples were purified by solid phase extraction.

##### Solid Phase Extraction for Sample Purification

Solid phase extraction (SPE) experiments were performed for the digested samples from the Ambi/urea extracts in order to purify and enrich analytical samples (peptides) prior the MS/MS analysis. Briefly, columns containing 300 mg of C18 material (Chromabond^®^ sorbent C18 ec, Macherey-Nagel GmbH &CO. KG, Düren, Germany) were activated with 6 mL of buffer A (50% ACN/50% distilled water containing 0.1% formic acid). A total of 6 mL of distilled water was then run for conditioning the solid phase. Samples were applied on the columns and thereafter, 6 mL of distilled water was run as the washing step. Peptides were finally eluted using 1 mL of buffer B (100% acetonitrile containing 0.1% formic acid) and the eluates were filled up to 5 mL with distilled water by giving the water through the columns. After that step, 100 µL of each sample was mixed with 10 µL of the internal standard and transferred to the vials for the analysis.

##### Mass Spectrometric Analysis

The MRM method developed and validated in the first part of this work using the C/M extraction was used without any modification to analyze the samples from the Ambi/urea extraction.

#### 2.2.9. Statistical Interpretation

Statistical evaluation was performed using two-way ANOVA in Graph Pad Prism (Version 8. 4.3; Northside Dr., San Diego, CA, USA—supplied by STATCON GmbH, Witzenhausen, Germany). Statistical significance was present if *p* < 0.05.

## 3. Results and Discussion

At the beginning it should be pointed out that we put more emphasis on the extraction of the ATIs using the chloroform/methanol (C/M) method with the intention of getting more or less a relatively pure crude extract in order to perform tests which would be not be possible while applying the options using urea. According to published literature, the inhibitory activity is also related to partial tendency of ATIs to quaternary structures (homodimeric or heterotetrameric forms) [15]—whereafter using urea would destroy such protein-protein interactions, thereby eventually diminishing the anticipated inhibition effect. The partial purity of the CM extract was established by the corresponding SDS-PAGE analysis, as shown in the following sections. This part was relevant for qualitative characterization (amylase inhibition) and for initiating the HPLC-MS/MS method development.

### 3.1. Intact Protein Analysis

#### 3.1.1. SDS PAGE and Analysis of the Relative Band Intensities

**Figure 3:**
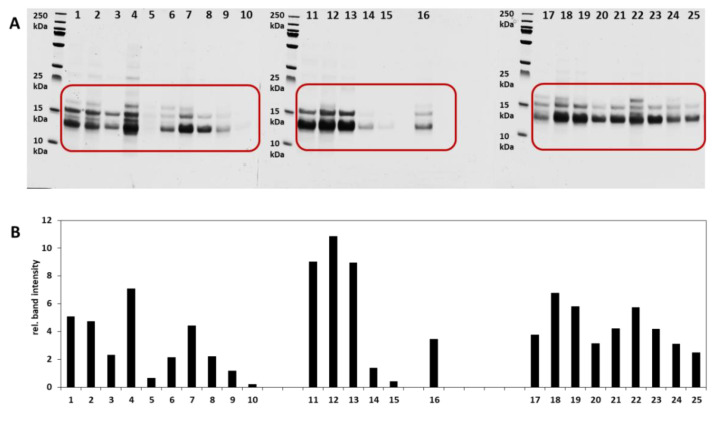
Sodium dodecyl sulfate-polyacrylamide gel electrophoresis (SDS-PAGE) with Coomassie Brilliant Blue R-250 staining of German wheat samples (**A**) and band intensity as relative protein content of the extracted ATIs (**B**). The marked region includes the bands between 10–20 kDa. 1–5; 6–10, and 11–15 represent the fractions whole-meal flour, centrifuged flour, passage flour, grain bran, and sling bran of the *Julius*, *Ponticus*, and *Elixier* cultivars, respectively. Samples 16 to 25 represent the extracts from *A. bayernkönig*, *Julius 2017IRGT reformIFindus*, *Nordkap*, *Patras*, *Ponticus 2017*, *Kerubino*, *Tobias*, and *Capo*, respectively.

**Figure 4:**
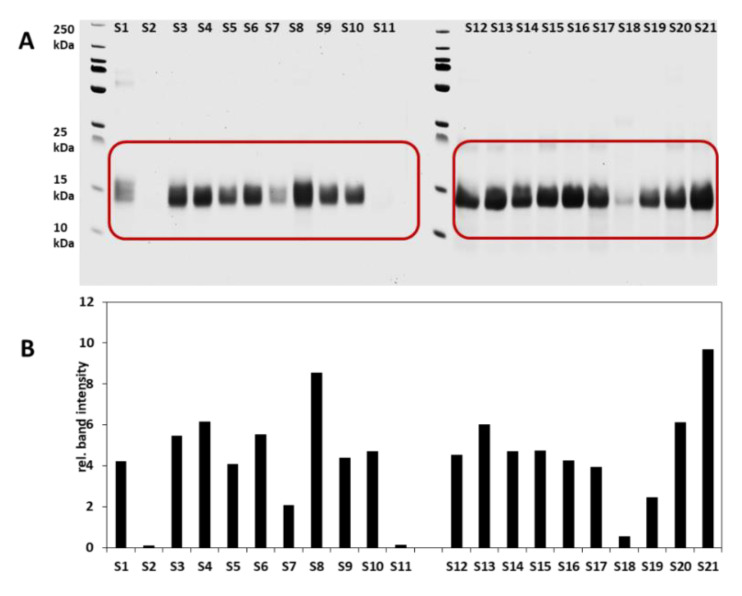
SDS-PAGE with Coomassie Brilliant Blue R-250 staining of Turkish wheat samples (**A**) and band intensity as relative protein content of the extracted ATIs (**B**). S1-S21 represent *Kunduru*, *Siyazan*, *Tosunbey*, *Tosunbey*, *Esperya, Kayra*, *Sivas 111/33*, *Ceyhan-99*, *Ceyhan-99*, *Pehlivan*, *Siyazan*, *Russian*, *Esperya*, *Ceyhan-99*, *Russian*, *Pehlivan*, *Bezostaja*, *Siyazan*, *AK-702*, *Esperya*, and *Altay* cultivars, respectively.

SDS-PAGE is an analytical method for separating proteins according to their molecular weight. The objective here was to initially visualize the composition and purity of the ATIs extracted by the CM mixture. Chloroform/methanol method was used to specifically extract protein fractions from the ATI family. To demonstrate this hypothesis, the qualitative analysis of extracted ATIs from 25 German and 21 Turkey wheat samples as well as their MW determination was performed using the SDS-PAGE under the reducing conditions. The results are presented in Figure 3 and Figure 4. The marked areas including bands between 10 and 20 kDa were used to determine the intensity related to the relative content of ATIs. From investigated German wheat samples, the *Elixer* fractions had a higher content of ATIs when compared to other cultivars. *Ponticus* and *Elixer* wheat cultivars debited similar protein band distribution pattern, in which the centrifuged flour had the highest relative protein content, followed by the whole-meal flour and the passage flour. The grain bran and sling bran fractions showed a relatively low content. The *Julius* wheat cultivar presented a different pattern. The grain bran fraction showed the highest relative number of protein band intensity, followed by the whole-meal flour and the centrifuged flour (Figure 3B). The comparison of the protein bands of the whole grain samples showed that the *Elixer* wheat had the highest overall relative intensity value (9.02). The other samples ranged from 1.74 (*Capo*) to 6.78 (*RGT Reform*). Statistical analysis revealed a significant difference in the ATI distribution in all analyzed samples according to the SDS-PAGE results (*p* < 0.05). Figure 4A,B presents the SDS-PAGE and relative band intensities of Turkish samples. From these results, it can be seen that samples exhibited different relative band intensities in the range of 10–20 kDa. Samples S2, S11, and S18 presented relatively low intensities (intensity values of 0.12, 0.14, and 0.55 for S2, S11, and S18, respectively), while S21 (9.68) and S8 (8.54) revealed higher intensity values. Finally, it can be concluded that SDS-PAGE delivers a fine tool to monitor the purity of the specific ATI extraction, as provided by C/M method. The vital step in the extraction scheme is the salting out effect in the last stages (Supplementary Figure S2), which effectively removes the other co-extracted proteins.

#### 3.1.2. Protein Contents According to Lowry

Protein contents of ATI extracts were also analyzed using the Lowry method. Each extract was measured in triplicate and the exemplary results expressed as mean values ± standard deviation (Supplementary Table S4A). According to these analyses, the passage meal fractions of German cultivars showed lower protein contents in *Julius* (0.45 µg/mg), *Ponticus* (0.54 µg/mg), and *Elixer* (0.52 µg/mg). Higher protein content was found in the *Julius* grain bran fraction (1.19 µg/mg), followed by the centrifuged flour fractions from *Ponticus* and *Elixer* cultivars (1.10 and 1.06 µg/mg, respectively). The protein content in the whole-meal flour for all varieties ranged between 0.63 and 0.95 µg/mg, while those of sling bran were of 0.75, 0.81, and 0.94 µg/mg for *Elixer*, *Ponticus*, and *Julius* cultivars, respectively. Statistical analysis revealed that there were no significant differences (*p* > 0.05) between the whole-meal flour, the centrifuged flour, and the sling bran (*Julius*), the grain bran and the sling bran (*Ponticus*). Protein contents of the fractionated samples were related to the protein content of the whole grain using the proportion of the fraction in the whole grain (Supplementary Table S5) and expressed as µg of protein per mg of sample. Setting the whole-meal flour samples as 100%, relative protein content of wheat fractions was estimated and lower values of 7%, 9%, and 14% proteins were obtained from the centrifuged flour fraction of *Julius*, *Elixer,* and *Ponticus* cultivars, respectively. Protein ratio of the passage flour fraction varied from 63% to 67%, and thus increased the relative protein content of this fraction. Those of the grain bran and sling bran fractions ranged from 15% to 39% and 19% to 21%, respectively.

Protein content of wheat cultivars *Julius* 2017, *RGT Reform*, *Findus*, *Nordkap*, *Patras Ponticus* 2017, *Tobias*, *Capo*, and *Kerubino* and the old wheat cultivar *Ackermanns Bayernkönig* were also analyzed, and overall, the average protein content of A-wheat cultivars (0.59 µg/mg) was slightly higher than that of E-wheat cultivars (0.50 µg/mg) (Supplementary Table S4). Within the A-wheat cultivars, *Julius* and *Tobias* had the lowest protein content (0.51 µg/mg). Protein content from *Patras* and *Findus* was about 0.6 µg/mg, while *RGT Reform* debited a higher value (0.66 µg/mg). The E-wheat cultivars *Capo* and *Kerubino* presented lower protein content (0.38 and 0.4 µg/mg, respectively), while *Tobias* and *Ponticus* values were 0.52 and 0.62 µg/mg, respectively. The old wheat cultivar *Ackermanns Bayernkönig* showed a protein content of 0.59 µg/mg.

Corresponding protein contents of the extracts for the Turkish samples ranged from 0.5 to 2.4 µg/mg flour with the CM method. The highest amount of protein could be extracted from the sample S10 (a hard wheat cultivar from central Anatolia, type “*Pehlivan*” from the year 2016). The lowest amounts were extracted from the samples S1, S11, and S19. The results also indicate that the soft wheat cultivar “*Siyazan*” has low protein content in the extracts (S2, S11, S18), but the content seems to be slightly affected by the region where it was cultivated. These results give the first impression of the amount of ATIs eventually present in the different samples analyzed. Protein contents of wheat samples from Turkey using option 2 of the Ambi/urea method ranged from 47.558 ± 1.337 µg/mg flour (S21) to 84.874 ± 2.743 µg/mg flour (S11). Conversely to the CM extraction, a higher amount of protein was extracted from samples S11 with the Ambi/urea buffer. It is well established that urea has a profound effect on the protein stability and structure; exerting the protein denaturation either directly by binding to the protein or indirectly by altering the solvent environment [34]. The result is a maximization of the yield of total extracted proteins. The effectiveness of the CM extraction method is relatively low (a short assessment is given in Supplementary Table S4A), but does allow an approximate evaluation of the biological activity. The content in mg/mL in both extracts has also been provided in Supplementary Table S4B.

### 3.2. Amylase and Trypsin Inhibition

**Figure 5:**
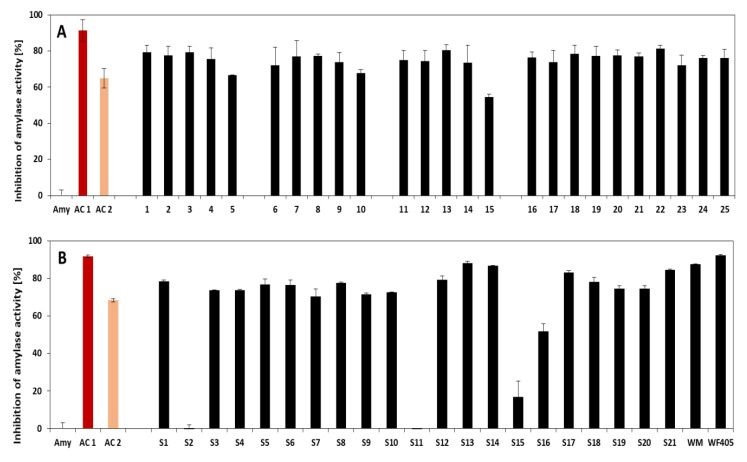
Inhibition of activity by ATIs extracted from German (**A**) and Turkish (**B**) wheat cultivars. Amy = negative control without ATI extract, AC = positive control with acarbose (1 mM and 50 µM). See Table 1 and Table 2 for sample allocation. *n* = 3. 1–5; 6–10, and 11–15 represent the fractions whole-meal flour, centrifuged flour, passage flour, grain bran, and sling bran of the *Julius*, *Ponticus*, and *Elixier* cultivars, respectively. Samples 16 to 25 represent the extracts from *A. Bayernkönig*, *Julius 2017*, *RGT reform*, *Findus*, *Nordkap*, *Patras*, *Ponticus 2017*, *Kerubino*, *Tobias*, and *Capo*, respectively. S1-S21 represent *Kunduru*, *Siyazan*, *Tosunbey*, *Tosunbey*, *Esperya*, *Kayra*, *Sivas 111/33*, *Ceyhan-99*, *Ceyhan-99*, *Pehlivan*, *Siyazan*, *Russian*, *Esperya*, *Ceyhan-99*, *Russian*, *Pehlivan*, *Bezostaja*, *Siyazan*, *AK-702*, *Esperya*, and *Altay* cultivars, respectively.

The DNS method was used to determine the inhibitory effect of extracted ATIs on porcine α-amylase activity. The activity of amylase without incubation with the ATI extract represents here 100% activity (accordingly 0% inhibition) and was used as reference to calculate the relative inhibition. Acarbose solutions (50 µM and 1 mM) were used as positive controls with corresponding inhibitory activities of 65 ± 3 and 91 ± 2%, respectively. The results are presented in Figure 5. As shown, inhibition of porcine α-amylase was detected in all samples and ranged from 60% to 80% for German and Turkish wheat cultivars, with exceptions for Turkish cultivars S2 and S11 that exhibited almost no inhibitory activity.

Comparing the inhibition activity with the results of the protein content (Lowry), it appears that the amount of extracted proteins (ATI-related) did not have direct influence on the inhibition activity. A possible partial denaturation during the CM extraction could explain the observed effect, but this assumption needs to be substantiated. It was not possible to clearly differentiate samples according to their inhibitory effects. More than the amount of proteins, the composition of individual extracted ATIs in each sample seems to be more decisive for determining the inhibitory effect. Gomez et al. [35] showed that inhibition increased with the amount of ATIs when only certain combinations of tetrametric inhibitor subunits were present and the different heterotetramers formations may in turn lead to differing activity of these inhibitors.

Furthermore, previous studies revealed that ATIs reacted differently depending on the type of alpha amylase—monomeric and heterotetrameric inhibitors being mostly active against insect α-amylase, while homodimeric inhibitors were more active against both insect and mammalian saliva α-amylases [36]. This suggests that using insect α-amylase instead of porcine α-amylase could allow better differentiation between samples with respect to their inhibitory activity. Finally, enzyme kinetics can be pointed out as an important feature to consider for future studies. According to the type of inhibition, the inhibitors may differently affect the kinetic parameters (maximum velocity and Michaelis constant). In the presence of high inhibitor concentrations as presented here, the saturation of enzyme active sites is quickly reached, and thus, does not allow a better differentiation of the activities. This was probably the case since the inhibition incubation was conducted during 10 min. The results of the positive control using acarbose showed that with a concentration of 50 mM, the amylase inhibition reached 65%. At a concentration of 1 mM (20 times more concentrated), acarbose debited an inhibition of 91%. Analysis at intermediate acarbose concentrations would have revealed the saturation step reached. It would therefore be advisable in future work to characterize and model the activity kinetics of each member of the wheat ATI family. All the same, the intention here was to primarily show that the ATIs can retain their inhibitory function while applying partial purification of the CM method.

Trypsin inhibition was also analyzed and the results are presented in Figure S5 (see supplementary data). The data again indicate that the trypsin inhibition is at least partly retained while applying the CM extraction procedure. Azocasein was used as substrate and soybean trypsin inhibitor (STI) at different concentrations was used as positive test. It appeared that trypsin had a strong activity that was not efficiently inhibited. STI at a concentration of 1 mg/mL showed an inhibition activity of 28%. Samples S2 (1.3%), S11 (1.2), S14 (2.4%), and S18 (2.3%) exhibited low inhibition activities, while higher inhibitions of 16.8%, 14.4%, and 12.6% were recorded with samples S1, S4, and S6, respectively. Overall, inhibition activities ranged under 20%.

The relatively low trypsin inhibition effects observed (compared to those of α-amylase inhibition) can also be related to the type of interaction with the protease used in this study. However, as disulfide bonds were reported to be involved in the α-amylase inhibitory effect of ATIs, the same disulfide bond formation in ATIs may also be involved in the protease inhibition. It has been reported that STI used as a positive test are relatively small molecules, with molecular weight between 7 and 9 kDa, rich in cysteine residues (20%) [37,38]. These cysteine residues form seven disulfide bonds that produce tertiary/quaternary structures, which are involved in the trypsin inhibitory activity of STI. Further work with different types of proteases and optimization of the procedure is being conducted and will be reported accordingly. Principally, these results give us the information that the following tryptic digestion to produce peptides for consequent identification and determining the relative abundance of ATIs while using the following HPLC-MS/MS would be possible.

### 3.3. LC-MS/MS Analysis

Elucidation of the distribution and relative quantification of the extracted wheat ATIs were performed by targeted tandem-mass spectrometry analyzes. For this purpose, a new MRM-based method was developed. A four amino acid peptide was used as internal standard for calibration and sample peak areas were normalized to 1 µg protein and to 1 mg weighed flour. The fraction-related protein proportions were used to determine the relative content of extracted ATIs from *Julius* 2018, Ponticus 2018, and Elixir fractions.

#### 3.3.1. Peptide Selection

Available protein sequences of 14 wheat ATIs (UniProt database) were used to develop the targeted MRM method. For each protein analyzed, peptide lengths of 5–25 amino acids were first analyzed and as the first test. The detected peptides were once more analyzed to improve and optimize the method parameters, and finally three to four peptides per protein with three to four transitions (fragments) for each peptide were selected for the final method. The selected peptides were allocated according to whether they were used as quantitative (biomarkers) or qualitative peptides. Three criteria led to the selection of the quantifier peptides: (1) the uniqueness (biomarkers were selected on the basis of their specificity); (2) high, robust, and stable signal intensities of each biomarker fragment; and (3) while applying the first two conditions and when several possible biomarkers were available, non-cysteine containing peptides were selected (reduction and alkylation reactions during the sample preparation affecting directly the cysteine residues). A summarizing list of the specificity of the peptides can be found in the Excel file “Peptide selection” in the Supplementary Information provided—it is noted that the specificity was primarily re-checked only for wheat, since this was the requirement defined by our objectives.

The final selection and assignment of the ATI peptides used for the relative quantification and qualification in this work are documented in the Supplementary Table S3. Similarity to other entries in the protein database UniProt is documented by alignment of the sequences in the Supplementary Information “selection of peptides” provided as an excel document. The optimized conditions for the multiple reaction monitoring (MRM) of the peptides and their transitions for the final HPLC-MS/MS method are documented in the Supplementary Table S6. The following biomarker peptides with their corresponding Q1-mass were selected to perform the relative quantification: K.VSALTGCR.A, R.TNLLPHCR.D, R.SDPNSSVLK.D, K.LTAASITAVCR.L, R.TSDPNSGVLK.D, R.YFMGPK.S, R.EQCVPGR.E, R.CEALR.V, R.ELAAISSNCR.C, K.LTAASITAVCK.L, R.NYVEEQACR.I were selected for ATIs P01083, P17314, P16850, P01084/85, P15851, P16159, P81496/Q43723/Q43691, P93602, P83207, Q4U199, and Q41540, respectively. Alternatives biomarkers were also found for three proteins P01083 (K.VPIPNPSGDR.A), P16159 (R.EVQMDFVR.I), and Q41540 (R.IEMPGPPYLAK.Q). These markers were then used as quantifier peptides to determine of the composition and the relative quantification of ATIs within the 46 analyzed samples.

#### 3.3.2. Method Validation

To investigate the method validation, the sample *Julius* (harvested in 2018) was used and consisted of determining the linearity of response, repeatability, and matrix effect.

##### Linearity of Responses

The linearity was performed for the IS in buffered blank (dissolved in distilled water) and the specific quantifier peptides in the sample *Julius* (harvested in 2018). Eight concentrations of extracted total protein of 50–2500 ng were measured in triplicate. The mean values of the peak area were plotted against the protein content. A calibration line was drawn from the measured values and the coefficient of determination was calculated. The results showed that for protein concentrations in the range of 50–2500 ng, all 11 quantifier peptides as well as the IS standard in buffered blank and in the wheat matrix exhibited a calculated *R*^2^ ≥ 0.993. For a successful validation the coefficient of determination should be at least 0.99. The graphs for linearity determination are presented as supplementary data (Figure S6, supplementary data).

##### Specificity

The specificity is given in the first instance by the uniqueness of the peptide allocated to a defined protein and analysis of its typical fragments as documented in the method part. The specificity against all the known entries of wheat was checked and is provided in the Supplementary Information in the Excel file “Peptide selection”.

##### Repeatability

The repeatability was determined exemplarily for the IS in buffered blank and the five selected specific peptides of the major ATIs in the sample *Julius* 2018 on two different days. The measurements were performed four times. For each fraction, average values and standard deviations were calculated to determine the relative standard deviation. Exemplarily and using the sample *Julius* 2018, relative standard deviations (inter day analysis) of 5.20, 4.43, 7.07, 3.66, 11.49, and 7.96 were found for the IS and quantifier peptides K.VSALTGCR.A (P01083), R.TNLLPHCR.D (P17314), R.SDPNSSVLK.D (P16850), K.LTAASITAVCR.L (P01084/85), and R.TSDPNSGVLK.D (P16851), respectively. Whole-meal flour, centrifuged flour, passage flour, grain bran, as well as sling bran fractions from *Julius* 2018 were also analyzed on two different days and the results of the measurements are summarized in Figure S7 (supplementary data). The relative standard deviations for inter-day analysis were calculated and ranged from 3.66 to 18.46. For intra-day analysis the standard deviation values were lower and also did not exceed 20%. Since all these results were under ≤20%, it allowed us to confirm the repeatability of the method.

##### Matrix Effects

The analysis of the IS in buffered blank and in the matrix sample of the five *Julius* 2018 fractions were evaluated. The measurements were performed four times and the average values and standard deviation were calculated. The concentration of IS in water was normalized to 100% and the rates in the other matrix samples were related to the normalized value. Figure S8 (supplementary data) documents the typical response for the peptide GWGG in the HPLC-MS/MS. Values of 89%, 80%, 78%, 87%, and 84% were registered for the IS in matrix samples whole-meal flour, centrifuged flour, passaged flour, grain bran, and sling bran, respectively. The results showed that for the internal standard GWWG for the Turkish wheat cultivars S1–S21, WM, and WF405 (allocation given in the materials section), the recovery rate was between 78% and 105% while applying the CM extraction method and the optimized final Ambi/urea extraction method. Some exemplary results of these measurements are given in Table S7 (supplementary data).

Additionally, matrix effect was also measured for the protein β-lactoglobulin (bovine, b-Lg, P02754-LACB_BOVIN) added to the extracted proteins prior to the digestion. The main aim was to monitor the digestion progress. The analysis was based on the response of the quantifier peptide K.GLDIQK.V in buffered blank as compared to that noted for the wheat samples. Figure S9 (supplementary data) illustrates the typical response for the peptide K.GLDIQK.V. The peak area of the peptide K.GLDIQK.V from the protein β-lactoglobulin added to the samples prior to the digestion ranged from 81% to 116% to that of the buffered blank, while applying the optimized final extraction method (Figure S9, supplementary data). An exception was observed for one sample (S11), where the value was under 70%. This indicates that in this sample, there were either strong protein/protein interactions presumably causing steric hindrance to the tryptic digestion, or there seems to be strong tryptic inhibition activity in the matrix of that particular sample. As already demonstrated, ATI extracts contained low trypsin inhibition activity, but it was assumed that here, firstly there was a high excess of trypsin, and secondly the application of the protocol documents the presence of urea up to 4M concentration, thus preventing the major secondary binding interactions between proteins. Some exemplary results of these measurements for both methods are given in Table S7. All the values obtained for optimized final Ambi/urea extraction method are illustrated in Figure S10 (supplementary data). In the absence of reference materials (matrixes and proteins) it was not possible to calculate the method recovery properly.

#### 3.3.3. Analysis and Relative Quantification of ATIs in German Wheat Cultivars

**Figure 6:**
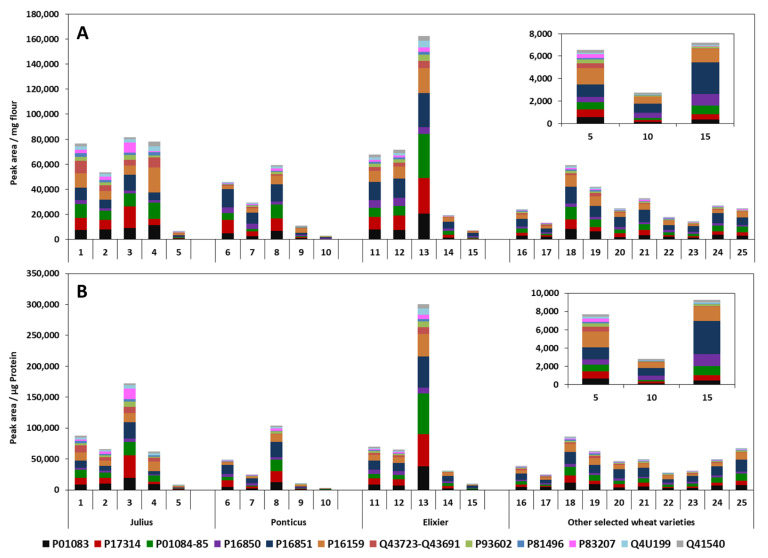
Distribution and relative abundance of ATIs from the German wheat cultivars presented as: (**A**) peak area obtained pro mg flour applied and (**B**) as peak area pro µg protein in the investigated CM extracts. Peak area was the response measured by the HPLC-MS/MS analysis for the quantifier peptide for the corresponding proteins. Side bar shows the sample allocation and the inset in each case shows the composition of the grain fraction “sling or spun bran”. 1–5; 6–10; and 11–15 represent the fractions whole-meal flour, centrifuged flour, passage flour, grain bran, and sling bran of the *Julius*, *Ponticus,* and *Elixer* cultivars, respectively. Samples 16 to 25 represent the extracts from *A. Bayernkönig*, *Julius 2017*, *RGT reform*, *Findus*, *Nordkap*, *Patras*, *Ponticus 2017*, *Kerubino*, *Tobias,* and *Capo*, respectively.

Figure 6 shows the distribution and relative abundance of ATIs from the German wheat cultivars presented as peak area obtained pro mg flour applied as well as peak area pro µg protein in the investigated CM extracts. Marker peptides peak areas were used for calculations. Significant differences in the relative ATIs content between cultivars were observed.

Comparing the investigated fractions (whole-meal four, centrifuged flour, passage flour, grain bran, and sling bran) from *Julius* 2018, *Ponticus* 2018, and *Elixer* cultivars, it appears that the passage flour fraction debited higher relative ATI contents for all the samples. The *Elixer* passage flour showed a value of 300,201.7 peak area/µg (PA/µg) protein, while values of 172,443.3 and 104,143.8 PA/µg proteins were registered for the passage flour of *Julius* 2018 and *Ponticus* 2018, respectively. Conversely, the sling bran fraction exhibited lower relative ATI contents. Statistical analysis showed a significant difference in ATI content of wheat fractions within each sample (*p* < 0.05). Looking at the distribution of the individual ATIs between the grain fractions, significant differences between fractions and cultivars were observed. The most common ATIs were P16851, P16159, P01083, P01084/84, P17314, and P16850. Samples contained 10–40% of each of these ATIs, with exceptions of proteins P83207 (10%) and Q41540 (11%) in *Julius* passage flour and *Ponticus* grain bran, respectively. The relative most common ATI in *Julius* was protein P16159, both the whole-meal flour (17%) as in the grain bran (17%) and the sling bran (28%). The dominant ATIs in centrifuged flour and in passage flour were P01083 (17%) and P17314 (22%), respectively. With *Ponticus* and *Elixer*, P16851 was the most represented ATI in the whole-meal flour (31%), centrifuged flour (31%), passage flour (24%), and sling bran (29%). ATI P16851 ranged from 22% to 40% in all *Elixer* fractions (Figure 6). Finally, as shown in the inset of Figure 6, the composition of the grain fraction “sling or spun bran” changes considerably with the ATI P16851 and P16159 becoming the main constituents.

From the other German wheat cultivars, *RGT Reform* (86,256.1 PA/µg protein), *Capo* (67,930.1 PA/µg protein), and *Findus* (62,951.9 PA/µg protein) showed higher amounts of ATIs, while only values of 28,259.3 and 24,863.2 PA/µg protein were obtained for *Ponticus* 2017 and *Julius* 2017, respectively (Figure 6). *Julius* and *Ponticus* are among the most important modern cultivars produced in Germany. The cultivar *Julius* is the most broadly cultivated soft wheat in the state of Brandenburg (19.1%) and the third most cultivated cultivar in Germany (5.5%) in 2018. It appears that these two cultivars contained only 28% (*Julius*) and 32% (*Ponticus*) of ATIs while comparing to the cultivar *RGT Reform*.

The distribution of the individual ATIs showed that as previously with the flour fractions, the relatively most common ATIs were P16851, P16159, P01084/85, P01083, P17314, and P16850. The proportion of each protein in all samples ranged from 7% to 34%. Protein P16851 with values between 21% and 34% was a major ATI in all wheat samples. ATI content of the old wheat cultivar *Ackermanns Bayernkönig* (39,031.1 PA/µg protein) was between the ranges of those of the modern cultivars. According to Dupont et al. [15], the relative distribution of wheat ATIs is as follows: 53% heterotetramers, 31% homodimers, and 16% monomers. The group of monomeric ATIs includes proteins of the 0.28 family (P01083). The homodimers consist of the ATIs 0.19 (P01085) and 0.53 (P01084), while the heterotetramers consist of a combination of CM1 (P16850), CM2 (P16851), CM3 (P17314), CM16 (P16159), and CM17 (P16852/Q41540) [7,17]. The results show that the group of heterotetramers ATIs was the most detected in all samples, followed by the homodimers and monomers. Geisslitz et al. [25] have determined the proportion of six ATIs in different wheat varieties by means of targeted quantitative LC-MS/MS and found that the most detected ATIs were 0.19 (P01085) and 0.53 (P01084), followed by CM3, CM16, 0.28 (P01083), and finally CM2. Feng et al. [36] found that ATI 0.19 was one of the most common ATIs present in wheat [30].

It appears that ATIs distribution in cultivars differed between the years. For example, the predominant ATIs in the *Julius* 2017 were P16851, P01083, and P16159, while in *Julius* 2018 the most abundant ATIs were P16159, P01084/85, and P17314. The same behavior was observed with *Ponticus*. The relatively most abundant ATIs were P16159, P16851, and P01084/85 in the sample of 2017; and P16851, P17314, and P01083 in the sample of 2018. Moreover, it was observed that relative content of the total ATIs of *Julius* and *Ponticus* differed between the years. Analysis revealed that the differences were statically significant (*p* < 0.05). ATI contents were found to be lower in the 2017 sample than those of 2018. *Julius* and *Ponticus* showed increases of 24,863.2 to 87,477.1 and 28,259.3 to 48,919.2 PA/µg protein from 2017 to 2018. Prandi et al. [24] analyzed ATI CM3 in durum wheat from different growing regions and cultivars and concluded that there were significant differences between the growing regions as well as between the years of harvest. This suggests that genetic factors, but also environmental factors such as climatic conditions influence the ATI content in the wheat grains.

#### 3.3.4. Analysis of Turkish Wheat Cultivars: Comparison CM and Ambi/urea Extracts

**Figure 7:**
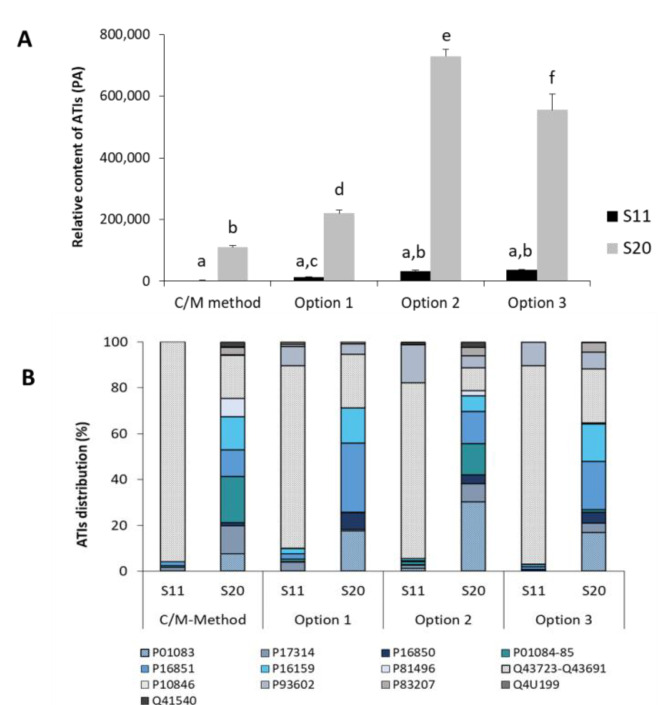
Relative ATI content (**A**) and ATI distribution (**B**) of wheat samples S11 and S20 from Turkey. The extraction was carried out using the C/M method and option 1–3 Ambi/urea method. Different letters mark significant difference (*p* < 0.05, ANOVA).

To improve the ATI extraction and thus their detection and quantification, we tried to extract ATIs from wheat samples using 100 mM ammonium bicarbonate buffer containing 4 M urea. Three different options of this method were tested. The comparative results from the analysis of the relative ATI contents and distribution in the extracts from Turkish wheat cultivars S11 and S20 are presented in Figure 7. Large differences in the relative content values were obtained from the different methods. If for the sample S11 (containing a low number of ATIs) there were no significant differences between the CM method and the three options of the Ambi/urea method, the behavior was different with the S20 sample, which contained a large number of ATIs. In fact, ANOVA analysis revealed that there were significant differences in the relative ATI content in the extracts obtained with the CM method and the three options of the Ambi/urea method (*p* < 0.05).

The three options of the Ambi/urea method exhibited higher amounts of relative ATIs compared to the CM method. Values of 210,000, 770,000, and 580,000 (PA) were registered for options 1, 2, and 3, respectively. Option 2 was found to have the highest value of the three options tested. The main differences between these options, particularly between options 2 and 3, were related to the reduction of disulfide bridges and alkylation of cysteine residues. It appears that proceeding sequentially by reduction followed directly by alkylation leads to a better sample preparation prior to the subsequent tryptic digestion step. The results indicate that simultaneous reduction/alkylation either during extraction of ATIs (option 1) or after extraction (option 2) do not give the envisaged high yield.

However, differences can be found not only in the total relative ATI content between the extraction methods, but also in the ATI distribution. For the wheat sample S11, the proteins Q43723-Q43691, which were detected together by mass spectrometry, were the dominant ATIs across all extraction methods. In the C/M extracts, these contributed to about 96% of the total ATI content, while P17314 (1.7%), P16851 (1.9%), and P01084-85 (0.8%) played only a minor role. In the extracts of the same wheat sample obtained with ammonium bicarbonate urea (option 1–3), almost all other ATIs can be detected (Figure S11, supplementary data). Wheat sample S20 showed a different distribution pattern compared to sample S11. The sample extracted by the C/M method was composed mainly of the proteins P01084-85 (20%), Q43723-Q43691 (19%), and P16159 (15%), which together account for approximately half of the total relative ATI content. In contrast, the protein P16851 was the dominant inhibitor with approximately 30% in the wheat sample S20 extracted with option 1, followed by Q43723-Q43691 (23%) and P01083 (18%). These proteins also account for approximately half of the total ATI content in option 2 and 3 extracts (Figure S11, supplementary data).

It can be concluded that the extraction solution not only has an influence on the ATI yield, but also on the distribution of individual ATIs in the total content. This fact allows the assumption that individual ATI types, possibly due to their structure (monomer, dimer, heterotetramer), can be extracted differently from the wheat samples.

**Figure 8:**
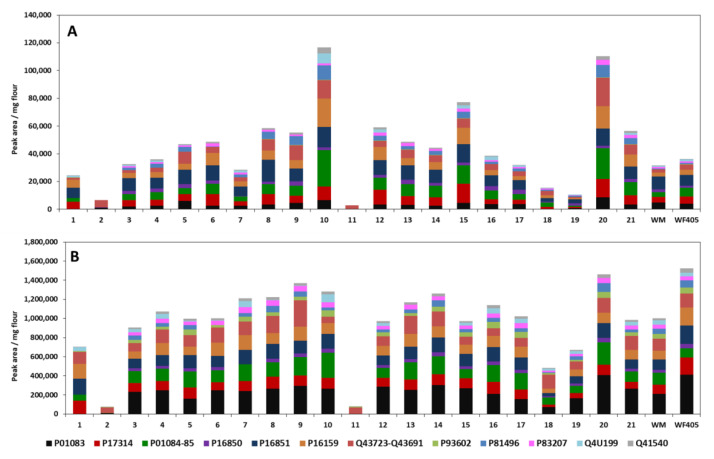
Distribution and relative abundance of the ATIs from Turkish wheat cultivars presented as: (**A**) peak area obtained pro mg flour applied in the investigated CM extracts of the samples and (**B**) in the option 2 Ambi/urea extracts. Peak area is the response measured by the HPLC-MS/MS analysis for the quantifier peptide for the corresponding proteins. WM and WF405 are commercially available wheat flours (WM = whole-meal flour, WF405 = extracted wheat flour type 405). See Table 2 for sample allocation.

Figure 8 indicates the distribution and relative abundance of the ATIs presented as peak area obtained pro mg flour applied in the investigated CM and option 2 of Ambi/urea extracts of Turkish samples. The first observation is that all wheat cultivars debited higher amounts of relative ATI contents with the Ambi/urea extraction compared to the CM extracts. Relative ATI abundance was correspondingly much higher in Ambi/urea extracts. For example, with the CM method, values of 33,884.1 ± 1815.6; 36,673.1 ± 751.9; 28,037.3 ± 580.6; and 10,396.6 ± 194.4 PA/mg wheat sample were obtained with cultivars S3, S4, S7, and S19, respectively. With the Ambi/urea method, values of 904,641.9 ± 4679.3 (S3); 1,072,944.6 ± 31,264.8 (S4); 1,210,032.7 ± 3324.1 (S7); and 671,254.9 ± 11,605.9 PA/mg wheat sample (S19) were obtained. The highest content can be detected for wheat sample S20, followed by sample S9 and sample S10. As with the C/M method extracts, the two wheat samples S2 and S11 provide the lowest relative ATI contents. The results also show that commercial wheat flour (type 405) has a significantly higher content than wheat flour from Turkey. Only between sample S20 and commercial flour was there no significant difference. In addition, commercially available whole wheat flour was analyzed by mass spectrometry, whereby this wheat flour had a 1.5-fold lower relative ATI content compared to wheat flour (Type 405). From these results, it is clear that option 2 of the Ambi/urea method is so far the best extraction method to achieve the relative quantification of wheat ATIs. Urea is, in principle, capable of denaturing proteins [39] and was also used in the study by Bose et al. [26] to extract ATIs from wheat. The results of our own work provide significantly higher ATI contents compared to the C/M method for all wheat samples extracted with option 2. The statistical analysis showed that, except samples for S2 and S11, the ATI content measured from all the samples was significantly different, while using either CM or Ambi/urea methods.

The second observation is the trend of comparison within the batch of the samples, which was different for the both extraction methods. In fact, samples S2 and S11 indicated lower measured ATI contents for CM and Ambi/urea methods; this was not the case for the other samples. Samples S7, S9, S17, and S19 showed comparatively lower amounts of ATIs with the CM method, while during Ambi/urea extract relatively higher contents in comparison to other samples were recorded. Conversely, sample S5 exhibited a higher content with CM method, while with the Ambi/urea extraction, its ATI content was in the average range compared to the other samples (Figure 8). These results can be directly related to the ATI composition extracted with both methods. In the C/M extracts, the protein P16851 accounts for a large part of the total ATI content, followed by P01084-85, P17314, and P01083, which together account for approximately 50–87%. Exceptions are the two wheat samples S2 and S11, in which only 2–4% of the proteinogenic inhibitors mentioned above are present. The above-mentioned predominant ATIs of the C/M extracts (P17314, P01084-85, P16851, P16159) contribute 3–51% of the total relative ATI content in the extracts obtained with option 2. In addition, P01083 is one of the most abundant ATIs of these wheat extracts. For example, looking at the samples S8 and S19, major ATIs extracted with the CM mixture were P16851 (27.3% and 28.0%, respectively), in the Ambi/urea extract it represented only 12.3% and 11.4% (for samples S8 and S19, respectively). The main ATI for both samples was protein P01083 (21.1% and 25.7% for samples S8 and S19, respectively). Again, the two samples S2 and S11 are an exception, in which Q43723-Q43691 are dominant (77% and 85%). The remaining ATIs in all wheat samples, except sample S11, take up only up to 6% of the total relative ATI content and thus play a minor role (Figure S11, supplementary data). The results of the mass spectrometric analysis show that the tetrameric forms mainly contribute to the total relative ATI content. In the literature there are also data available that deal with the percentage distribution. However, the analyzed ATIs in the literature partly differ from those of the own work (wheat cultivars), which makes a comparison rather difficult. Nevertheless, published values indicate that the heterotetrameric forms are the most common [15], which tends to agree with the results of our own analysis. This is especially true for the wheat extracts obtained with option 2. The data presented also reflect to some extent the observations of Geisslitz et al. [25], where 0.19 (P01085) and 0.53 (P01084) followed by CM3, CM16, 0.28 (P01083) were the major ATIs determined, which also belong to the dominant ATIs in the wheat samples of our own work.

Further, the two ATIs Q43723-Q43691, which were detected together by mass spectrometry, account for a not negligible proportion of the total ATI content. They contribute 7–96% (S1, S11) to the total ATI content in the C/M extracts and 5–85% (S10, S11) in the option 2 extracts (Figure S11, supplementary data). In the available literature, the proteinogenic inhibitors of the CMX1/CMX3 type could only be relatively quantified by Bose et al. [26]. For this reason, it would be desirable to extend the investigations in this respect. This is due to the fact that the two ATIs were present in significant amounts in the wheat samples from Turkey and, based on sequence alignments, an influence on the initiation of immune reactions via the mentioned proteins is suspected [22].

The analyzed wheat samples of the present study differ in their growing regions and also in their harvest years. When comparing the total relative ATI contents between the wheat extracts of the C/M method, the much higher values for the durum wheat samples S10 (cultivar *Pehlivian*) and S20 (cultivar *Esperya*) are noticeable (Figure 8A). Common to both samples is the growing region (Central Anatolia) and the harvest year (2016). The same durum wheat extracts obtained with option 2 also tended to have higher ATI contents than the other samples (Figure 8B). However, for both the C/M extracts and option 2 extracts, there were also differences noted between different wheat varieties from the same growing region (Central Anatolia) and the same crop year (2016) (wheat samples: S4, S10, S20, S21). In addition to the crop variety, the growing region also seems to have an influence on the total ATI content, as significant differences can be detected when considering the ATI content within a crop variety. The significance of both factors on the ATI content has also been described in the literature [24].

The cultivar *Siyazan* (wheat samples: S2, S11, S18) of different regions shows remarkably low values, whereas all three samples are assigned to the same wheat species, namely common wheat. In the already mentioned study by Geisslitz et al. [25], in which an absolute quantification of the ATIs was carried out by a specific LC-MS/MS-SIDA assay (stable isotope dilution assay, SIDA), no significant differences in the total ATI content could be detected between common wheat (*Triticum aestivum* L.) and durum wheat (*Triticum durum* L.). However, the results of their analysis provide partly significant differences of individual ATIs between the two wheat species. Characteristic for the cultivar *Siyazan* of our own work is the high proportion of ATIs Q43723-Q43691 (C/M extracts: 21–96%, option 2 extracts: 30–85%), which are thus the predominant proteins in these soft wheat samples (Figure S11, supplementary data). In the majority of the durum wheat samples, however, P16851 (C/M extracts) and P01083 (option 2 extracts) are the dominant ATIs. In contrast are the results of the analysis by Geisslitz et al. [25], in which CM2 (P16851) is one of the least abundant ATIs.

Finally, the comparison with published data therefore indicates that the extraction buffer system determines the ATI proteins extracted and the composition derived thereof but also depends on the wheat cultivar considered for the investigation.

Overall, it appears that CM-type ATIs (P16850, P16851, P17314, P16159, and P16852/Q41540) were predominant in the extracts from the CM mixture while ATIs type 0.28 (P01083), 0.19 (P01085), and 0.53 (P01084) represented the major fractions in the Ambi/urea extracts. Thereafter, we can observe that although the Ambi/urea buffer allowed the extraction of the majority of ATIS, type 0.19, the other ATI families, including the CM proteins, were also well extracted. The high amounts of ATIs relative content measured allowed us to claim that option 2 of the Ambi/urea buffer is the best method to accomplish extraction of ATIs from wheat cultivars for the subsequent analysis of their relative content.

## 4. Conclusions

This work was focused on the development of a comprehensive multiple reactions monitoring high-resolution tandem mass spectrometric based method for the simultaneous identification and relative quantification of wheat ATIs. A total of 25 German and 21 Turkish wheat cultivar samples were analyzed. Internal standard was used to calibrate and normalize the data and the application of β-latoglobulin allowed the digestion process to be monitored. Validation tools such as linearity, specificity, and effect of matrix allowed a primary test for the robustness of the proposed method. The developed method integrated analysis of all the 14 reviewed wheat ATIs currently listed by the online database UniProt database. In total, 11 individual biomarkers for proteins P01083, P01083, P17314, P16850, P15851, P16159, P16159, P93602, P83207, Q4U199, Q41540, Q41540 and two common biomarkers for proteins P01084/P01085 and P81496/Q43723/Q43691 were found. The developed method enabled the identification and relative quantification of ATIs in all or part of the 46 wheat cultivars analyzed. Chloroform/methanol mixture (2:1, *v/v*) was first used to develop the method to deliver relatively pure ATI mixtures. The further improvement of extraction was to make the analysis more comprehensive. Significant differences in terms of the composition as well as in the relative content emerged. Ammonium bicarbonate with its second variant presented much higher ATIs content compared to the chloroform/methanol extract. Finally, the developed LC-MRM-MS method allowed the analysis of the main commonly analyzed wheat ATIs as well as ATIs that have not been reported in the recently documented analytical methods. The method permits the analysis and encompasses all reviewed ATIs in wheat cultivars as well as wheat-based products in a single method. Corresponding synthetic peptides for the established biomarker will allow absolute quantification in the following studies. Further work is directed to testing the effect of processing on the response of individual ATIs.

## Figures and Tables

**Table 1:** Selected German wheat cultivars and their available data for the protein comparison.

Cultivar	Cultivation Location	Harvest Year	Quality *	Protein ** (g/100 g)
*Julius*	Münchenberg	2018	A	14.6
*RGT Reform*	Grünow	2018	A	14.7
*Findus*	Sonnewalde	2017	A	13.6
*Nordkap*	Sonnewalde	2017	A	12.4
*Patras*	Sonnewalde	2018	A	12.4
*Ponticus*	Sonnewalde	2018	E	15.7
*Kerubino*	Münchenberg	2018	E	12.5
*Tobias*	Angermünde	2018	E	14.0
*Capo*	Schmogrow	2018	E	11.3
*Elixer*	n.a	2018	C	12.8
*Ackermanns Bayernkönig*	Münchenberg	2018	X	19.4

n.a.—not available; * The German federal variety office (Bundessortenamt) allocates different varieties of common wheat according to their qualities. They are divided into the four quality groups E (elite wheat), A (quality wheat), B (bread wheat) and C (other wheat varieties), X (cross between wheat and spelt wheat). They differ in their volume yield, surface texture, and elasticity of the dough, falling number, sedimentation value, flour yield, and water absorption. ** Protein content in % as determined with KJELDAHL method.

**Table 2:** Selected Turkish wheat cultivars and their available data for the protein comparison.

Sample No	Cultivar	Cultivation Location	Harvest Year	Type	Protein * (g/100 g)
S1	*Kunduru*	Central Anatolia	2004	Hard	11.8
S2	*Siyazan*	Easthern Anatolia	2019	Soft	12.1
S3	*Tosunbey*	Central Anatolia	2019	Hard	13.0
S4	*Tosunbey*	Central Anatolia	2016	Hard	13.0
S5	*Esperya*	Central Anatolia	2019	Hard	14.3
S6	*Kayra*	Aegean Region	2018	Half hard	13.5
S7	*Sivas 111/33*	Central Anatolia	2018	Soft	12.7
S8	*Ceyhan-99*	Southeasthern Anatolia	2019	Hard	14.0
S9	*Ceyhan-99*	Blacksea Region	2019	Hard	14.0
S10	*Pehlivan*	Central Anatolia	2016	Hard	12.4
S11	*Siyazan*	Central Anatolia	2019	Soft	15.6
S12	*Russian*	Blacksea Region	2019	Hard	n.a
S13	*Esperya*	Marmara	2019	Hard	14.3
S14	*Ceyhan-99*	Southeasthern Anatolia	2016	Hard	14.0
S15	*Russian*	Mediterranean Region	2019	Hard	13.1
S16	*Pehlivan*	Marmara	2018	Hard	12.4
S17	*Bezostaja*	Central Anatolia	2019	Hard	12.0
S18	*Siyazan*	Blacksea Region	2019	Soft	11.8
S19	*AK-702*	Central Anatolia	2018	Soft	10.9
S20	*Esperya*	Central Anatolia	2016	Hard	13.9
S21	*Altay*	Central Anatolia	2016	Half Hard	11.8

n.a.—not available; * Protein content in % as determined with KJELDAHL method.

## Supplementary Materials

The following are available online at https://www.mdpi.com/2304-8158/9/10/1448/s1, Figure S1: Background on the milling processing, Figure S2: Extraction scheme for ATIs from wheat as reported in our previous study [1] using a chloroform/methanol (C/M) mixture in a ratio of 2:1, Figure S3: Selection of the protein, their peptide ions and transitions in Skyline (A), Response (peak area) in the three-part optimization process, Figure S4: Comparison of the three options used to perform the Ambi/urea extraction, Figure S5: Inhibition of trypsin by ATIs extracted from Turkish wheat cultivars, Figure S6: Linearity measurement of the specific ATI quantifier peptides and the internal standard (IS), Figure S7: Relative standard deviation from the determination of repeatability of the analysis for the grain fractions of the wheat variety *Julius* (2018), Figure S8: Chromatogram of the peptide GWGG, Figure S9: Chromatogram of the peptide K.GLDIQK.V from the protein beta-lactoglobulin added to the samples prior to the digestion, Figure S10: The recovery of IS (internal standard GWGG) and the quantifier peptide for b-Lg used to monitor the digestion (GLDIQK) in all the samples while applying the optimized final extraction method, Figure S11. Relative ATI distribution of wheat samples from Turkey carried out using (A) the C/M method and (B) the option 2 of Ambi/urea buffer, Table S1: Preliminary research on amylase/trypsin inhibitors from wheat (UniPtot—as of 15-02-2019), Table S2: Final selection of the known amylase/trypsin inhibitors (UniProt KB, 20.01.2020) analyzed in this work, Table S3: Final selection and assignment of the ATI peptides used for analysis, Table S4A: Protein content of the CM extracts from German wheat cultivars as determined by the LOWRY assay, Table S4B: Protein concentration of wheat samples from Turkey. The protein extraction was performed with a chloroform/methanol mixture (C/M method) and with an extraction buffer consisting of ammonium bicarbonate and urea (option 2), Table S5: Percentage of fractions of whole grain for the wheat varieties *Julius* 2018, Ponticus 2018 and Elixer (the data is based on the grinding protocol applied and documented in Supplementary Figure S1), Table S6: The optimized conditions for the multiple reaction monitoring (MRM) for the final HPLC-MS/MS method, Table S7: Summary of recovery tests for the CM method and the optimized final extraction method.
