# Supplementary material for: Comprehensive Characterization and Relative Quantification of α-Amylase/Trypsin Inhibitors from Wheat Cultivars by Targeted HPLC-MS/MS
Source: Foods. 2020 Oct 13;9(10):1448. doi: 10.3390/foods9101448 (PMC7600490; doi:10.3390/foods9101448)
Supplement: Supplementary file 1 [file foods-09-01448-s001.zip › Supplementary Data-12-10-2020.pdf]

Supplementary Material

Comprehensive characterization and relative quantification of α-amylase/trypsin inhibitors from wheat cultivars by targeted HPLC-MS/MS

Sorel Tchewonpi Sagu[1], Lynn Zimmerman[1], Eva Langräber[1], Thomas Homann[1], Gerd Huschek[2], Haydar Özpinar[3], Florian J. Schweigert[1] and Harshadrai M. Rawel[1,*]

1. University of Potsdam, Institute of Nutritional Science, Arthur-Scheunert-Allee 114-116, 14558 Nuthetal, Potsdam, Germany; sorelsagu@uni-potsdam.de (S.T.S.); lyzimmer@uni-potsdam.de (L.Z); e.landgraeber@live.de (E.L); homann@uni-potsdam.de (T.H.); fjschwei@uni-potsdam.de (F.J.S)

2. IGV-Institut für Getreideverarbeitung GmbH, Arthur-Scheunert-Allee 40/41, D-14558, Nuthetal OT Bergholz-Rehbrücke, Germany; gerd.huschek@igv-gmbh.de (G.H.)

3. School of Applied Science, Department of Gastronomy, İstanbul Altınbaş Üniversitesi, Büyükdere Cad.No 147, Esentepe İstanbul, Turkey; haydarozpinar@gmail.com (H.O)

* Corresponding author.

Tel: +49-33200-88-5525/5578 (H.R.)

E-mail: rawel@uni-potsdam.de

Content

Figure S1. Background on the milling processing

Figure S2. Extraction scheme for ATIs from wheat as reported in our previous study [1] using a chloroform/methanol (C/M) mixture in a ratio of 2:1. RT = Room temperature (25°C)

Figure S3. Selection of the protein, their peptide ions and transitions in Skyline (A), Response (peak area) in the three-part optimization process (Step A – determination of the retention time, Step B - optimization of the collision energy for the transitions and Step C- response under the final MRM conditions determined) by Skyline (B) and an exemplary spectrum of the transitions of the selected peptide ion (TNLLPHCR) (C)

Figure S4. Comparison of the three options used to perform the Ambi/urea extraction.

Figure S5. Inhibition of trypsin by ATIs extracted from Turkish wheat cultivars. Tryp = negative control without ATI extract, STI = positive control with soybean trypsin inhibitor (1 mg/ml). See Tables 1 and 2 for sample allocation.

Figure S6. Linearity measurement of the specific ATI quantifier peptides and the internal standard (IS)

Figure S7. Relative standard deviation (rel. SD) from the determination of repeatability of the analysis for the grain fractions of the wheat variety Julius (2018). The presented peak areas are related to corresponding 1 µg protein. n.s. = not significant

Figure S8. Chromatogram of the peptide GWGG (MS conditions are documented in the Supplementary Table S4)

Figure S9. Chromatogram of the peptide K.GLDIQK.V [8, 13] from the protein beta-lactoglobulin added to the samples prior to the digestion (MS conditions are documented in the Supplementary Table S4)

Figure S10. The recovery of IS (internal standard GWGG) and the quantifier peptide for b-Lg used to monitor the digestion (GLDIQK) in all the samples (S1-S21, WM and WF405, allocation given in the materials section) while applying the optimized final extraction method

Figure S11. Relative ATI distribution of wheat samples from Turkey carried out using (A) the C/M method and (B) the option 2 of Ambi/urea buffer. S1-S21 represent *Kunduru, Siyazan, Tosunbey, Tosunbey, Esperya, Kayra, Sivas 111/33, Ceyhan-99, Ceyhan-99, Pehlivan, Siyazan, Russian, Esperya, Ceyhan-99, Russian, Pehlivan, Bezostaja, Siyazan, AK-702, Esperya* and *Altay* cultivars, respectively.

Table S1. Preliminary research on amylase/trypsin inhibitors from wheat (UniPtot – as of 15-02-2019) - Data with calculated isoelectric points (pI) and molecular weight of selected ATIs and CM proteins (Wheat; 14 reviewed - Records with information extracted from literature and curator-evaluated computational analysis and 2 Unreviewed - computationally analyzed records that await full manual annotation)

Table S2. Final selection of the known amylase/trypsin inhibitors (UniProt KB, 20.01.2020) analyzed in this work = green; confirmed = *

Table S3. Final selection and assignment of the ATI peptides used for analysis; AA = Amino acid; Qual. = qualifier; Quant. = quantifier. Similarities to other entries in the database is documented by alignment of the sequences in the Excel file supplementary information “selection of peptides”

Table S4A. Protein content of the extracts from German wheat cultivars as determined by the LOWRY assay

Table S4B. Protein concentration of wheat samples from Turkey. The protein extraction was performed with a chloroform/methanol mixture (C/M method) and with an extraction buffer consisting of ammonium bicarbonate and urea (option 2).

Table S5. Percentage of fractions of whole grain for the wheat varieties Julius 2018, Ponticus 2018 and Elixer (the data is based on the grinding protocol applied and documented in supplementary Figure S1)

Table S6. The optimized conditions for the multiple reaction monitoring (MRM) for the final HPLC-MS/MS method; Q1 = Precursor mass; Q3 = Transition mass; CE = Collision energy

Table S7. Summary of recovery tests for the CM method and the optimized final extraction method (Section 2.2.1/2.2.7 in the manuscript)

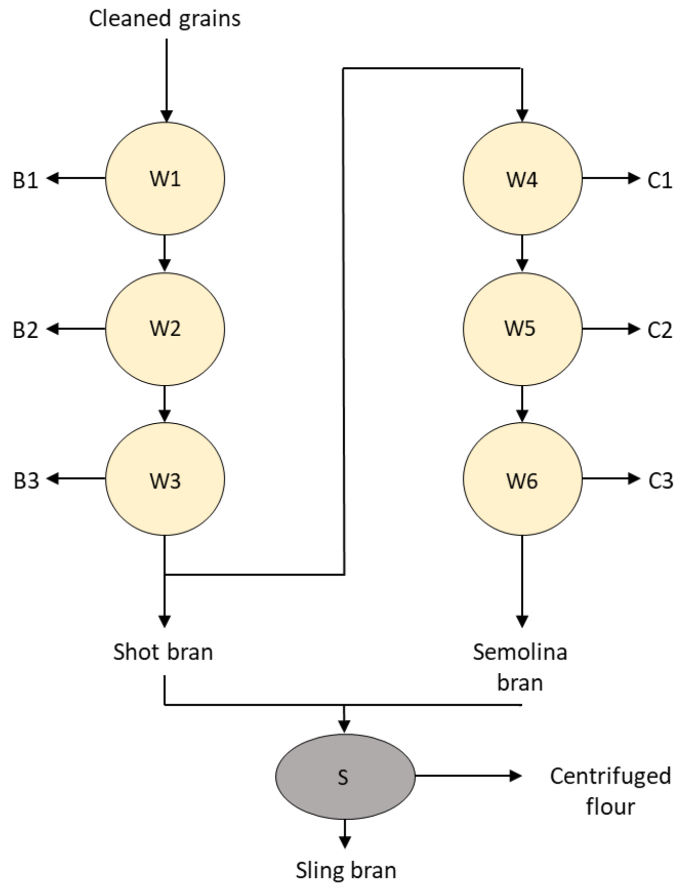

W = Grinding roller (1-3 Corrugated rolls, 4-6 fine fluted / Smoothing rolls);
B = Passage flour (shot passages);
C = Passage flour (Dissolution-/Grindingpassages);
S = Grain centrifuge.

Figure S1. Background on the milling processing

Figure S1 shows a schematic representation of the grinding process. The aim of the process is to achieve the cleanest possible separation of the endosperm from the outer layers of the grain kernel. The outer layers, mainly the fruit and seed husk and variable parts of the aleurone layer and the marginal layer of the endosperm are summarized as bran. Depending on the processing, the germ is separated from the endosperm or is combined together with the bran. The bran fractions used in this paper also contained the germ. The cleaned grain is broken up by the rollers and the endosperm is separated from the outer layers of the grain by the increasingly fine rollers. The flour is separated from the coarse outer layers by sieving and collected as fractions of passage flours B1-B3. After these first three rolling processes, the shot bran containing the coarse outer layers, the seedling and parts of the aleurone layer is removed. The remains of the grain kernel are passed on to finer rollers, where parts of the aleurone layer are removed from the last shell remains and sieved off with the passage flours C1-C3. The semolina bran remains. The semolina bran and shot bran (together they make up the grain bran) are spun in a grain centrifuge. Residues of the endosperm and the aleurone layer are separated from the husks and the germ. This produces the spun (sling) bran, also known as centrifugal bran, and the separated flour, known as centrifuged flour.

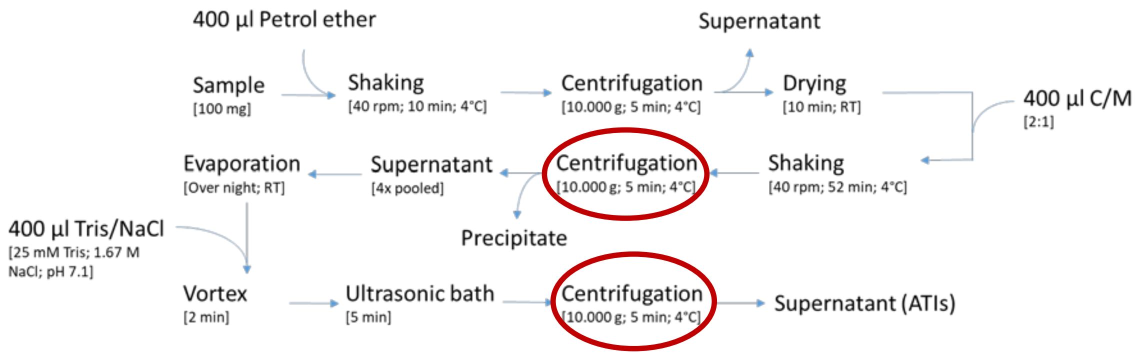

Figure S2. Extraction scheme for ATIs from wheat as reported in our previous study [1] using a chloroform/methanol (C/M) mixture in a ratio of 2:1. RT = Room temperature (25°C)

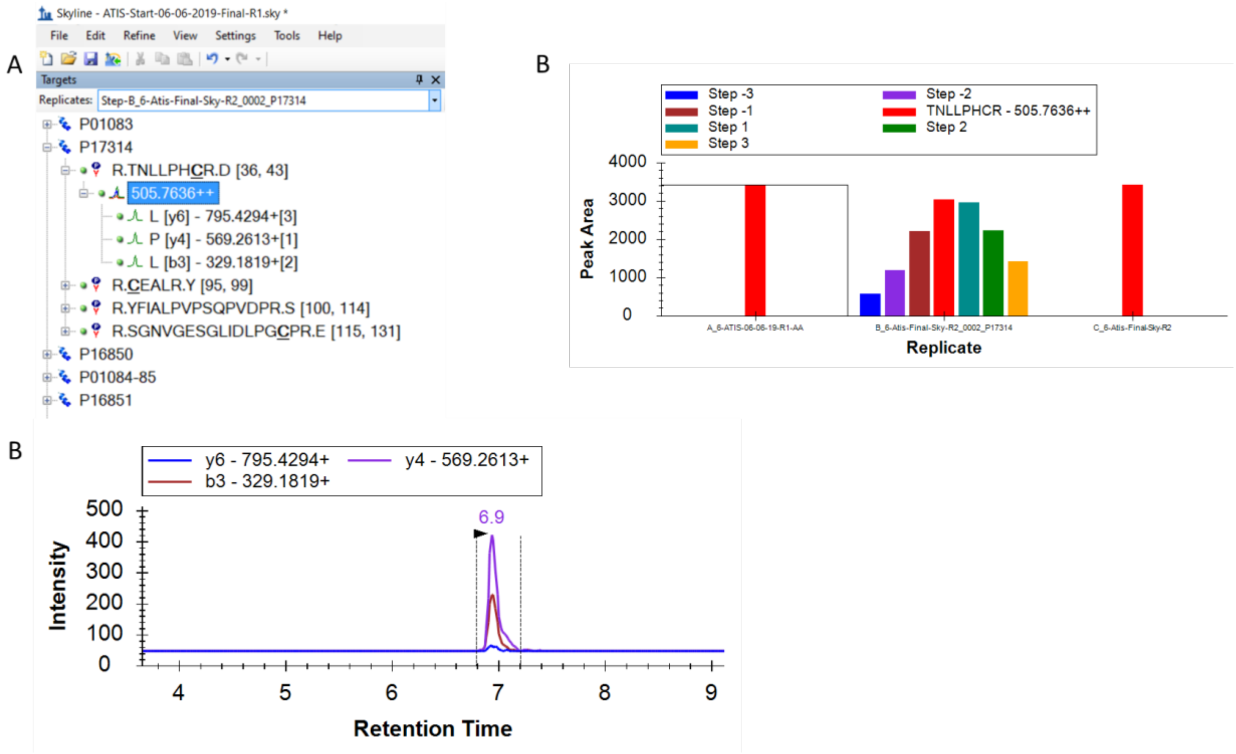

Figure S3. Selection of the protein, their peptide ions and transitions in Skyline (A), Response (peak area) in the three-part optimization process (Step A – determination of the retention time, Step B - optimization of the collision energy for the transitions and Step C- response under the final MRM conditions determined) by Skyline (B) and an exemplary spectrum of the transitions of the selected peptide ion (TNLLPHCR) (C).

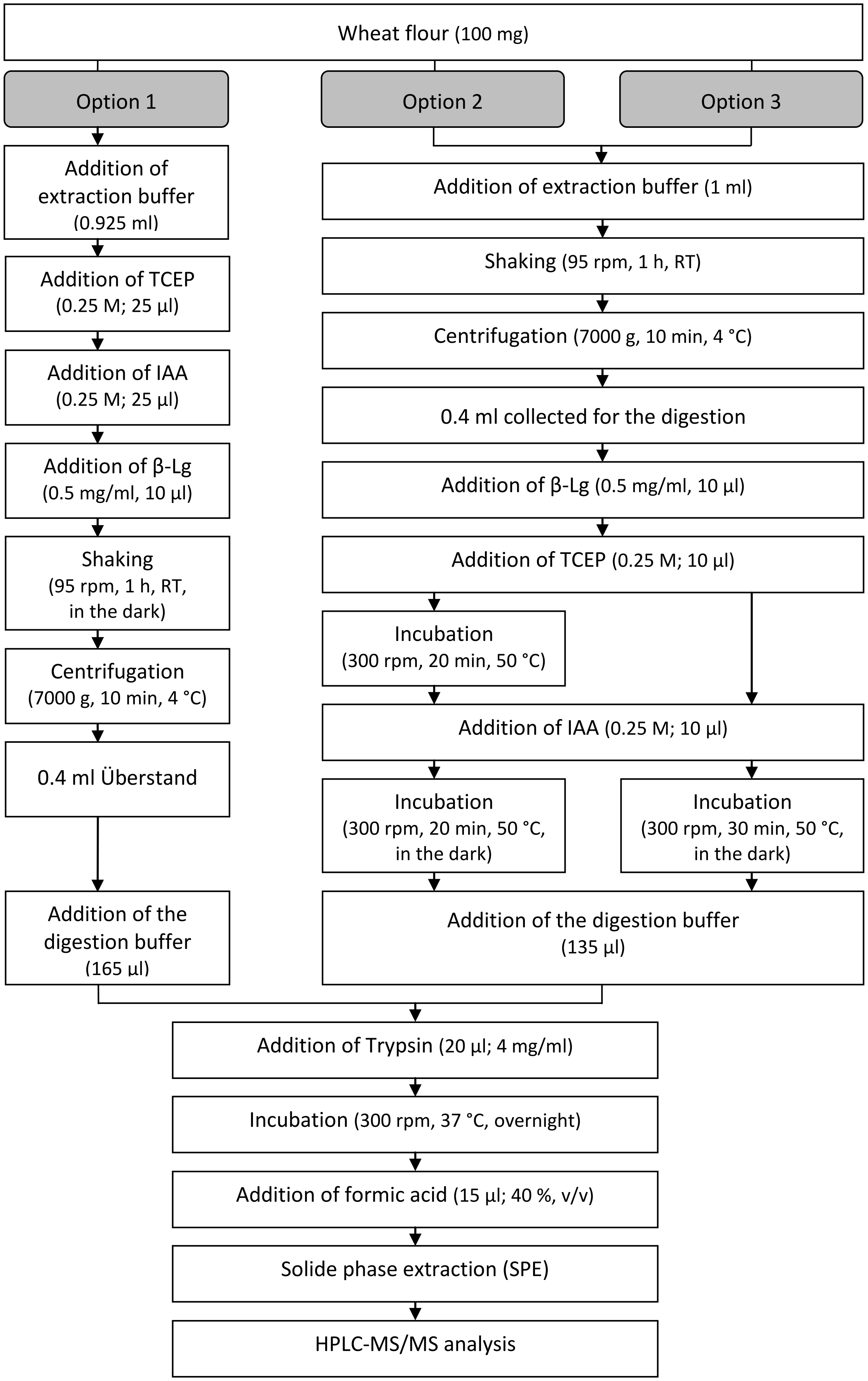

Figure S4. Comparison of the three options used to perform the Ambi/urea extraction.
IAA: Iodoacetamid, β-Lg: β-Lactoglobulin, TCEP: Tris-(2-carboxyethyl)-phosphin Hydrochlorid

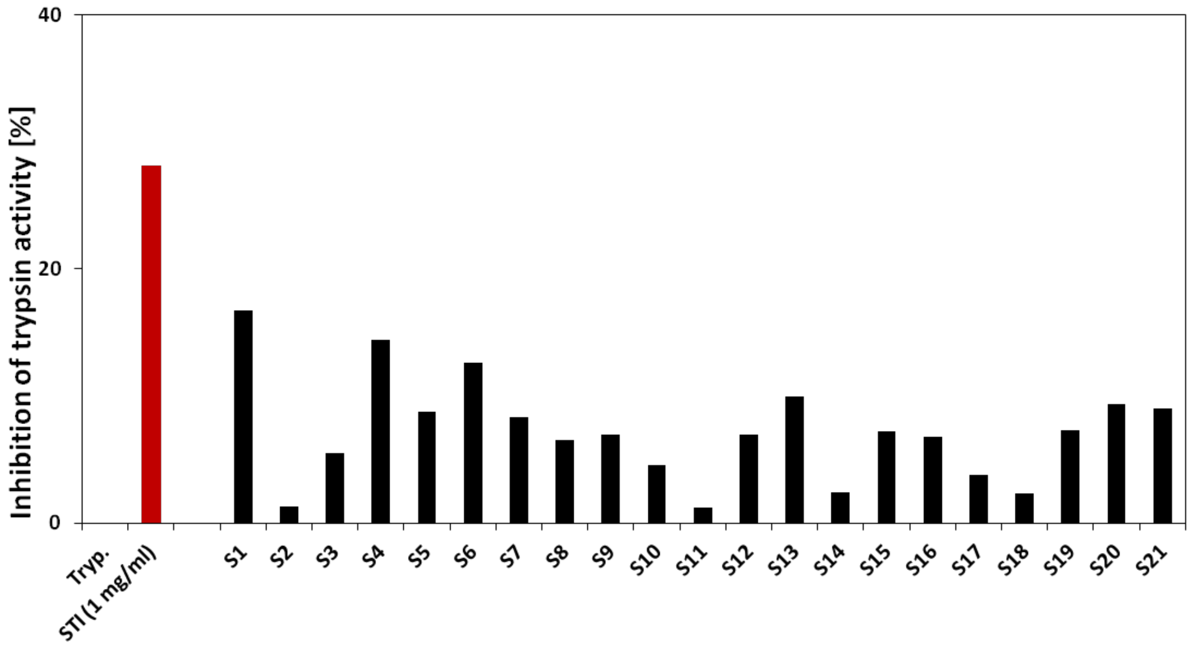

Figure S5. Inhibition of trypsin by ATIs extracted from Turkish wheat cultivars. Tryp = negative control without ATI extract, STI = positive control with soybean trypsin inhibitor (1 mg/ml). See Tables 1 and 2 for sample allocation

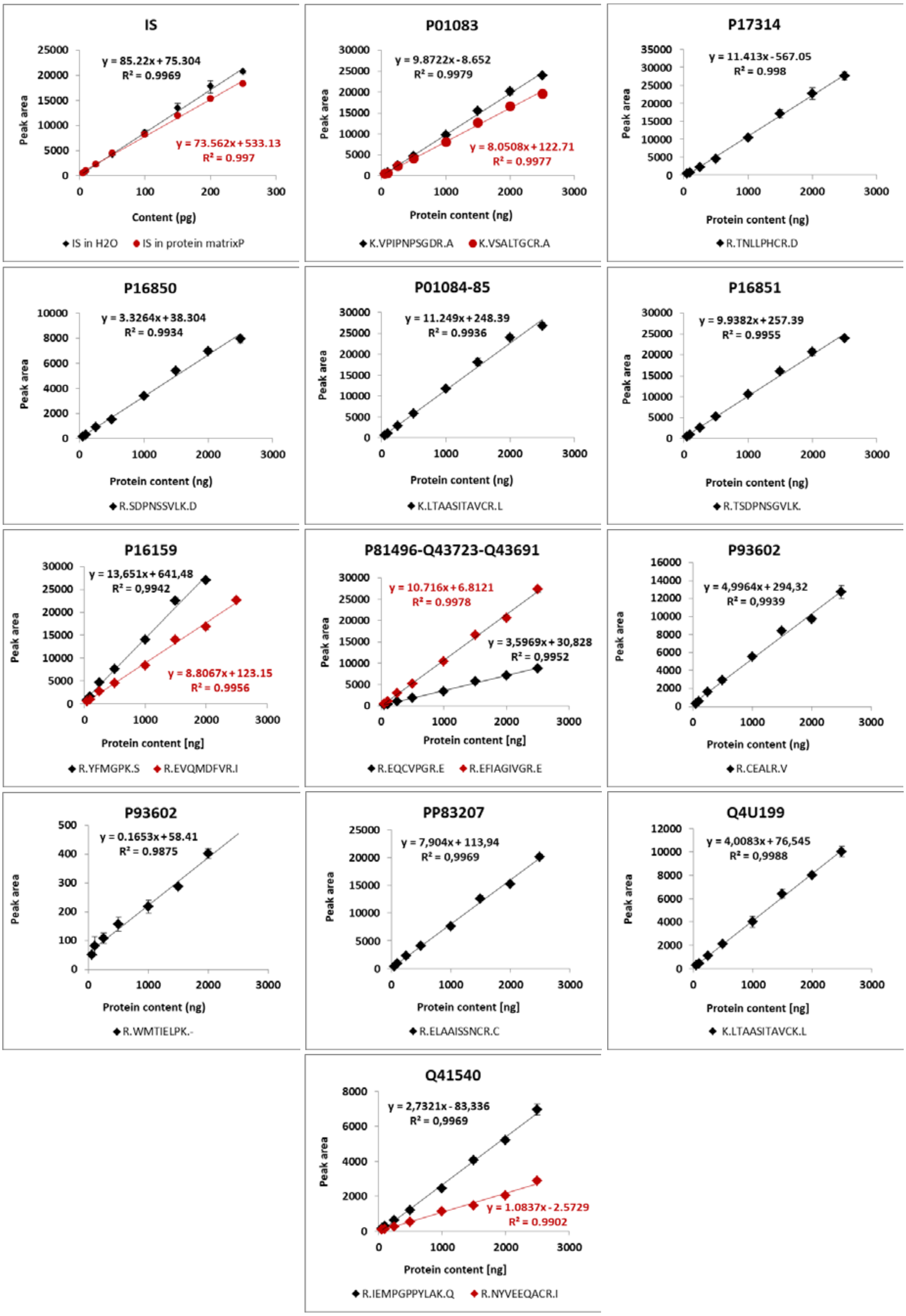

Figure S6. Linearity measurement of the specific ATI quantifier peptides and internal standard (IS)

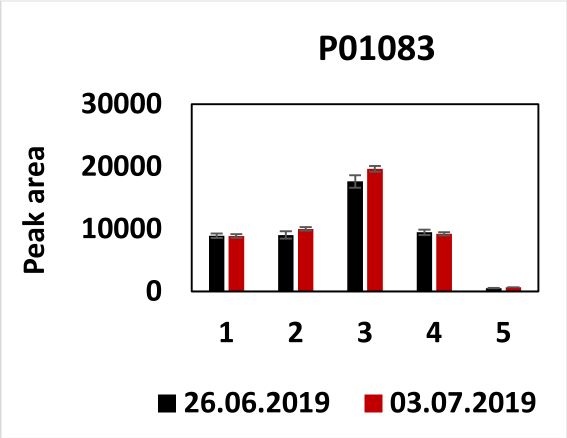

Nr.	t-Test	Significance	Mean	SD	Rel. SD
1	0.898	n.s.	8912.81	394.82	4.43
2	0.056	n.s.	9553.39	745.44	7.80
3	0.068	n.s.	18646.75	1604.96	8.61
4	0.463	n.s.	9367.05	398.58	4.26
5	0.044	n.s.	628.67	86.57	13.77

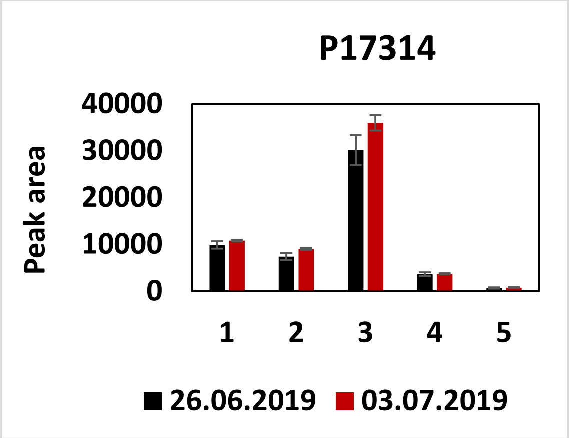

Nr.	t-Test	Significance	Mean	SD	Rel. SD
1	0.055	n.s.	10352.28	732.22	7.07
2	0.006	**	8234.02	996.81	12.11
3	0.025	*	33472.16	3810.23	11.38
4	0.633	n.s.	3699.31	248.82	6.73
5	0.318	n.s.	759.65	106.75	14.05

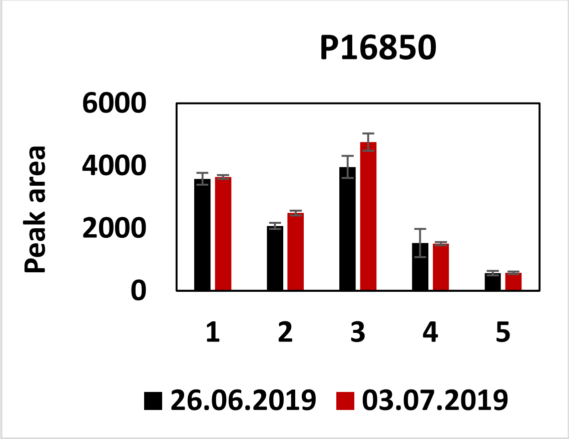

Nr.	t-Test	Significance	Mean	SD	Rel. SD
1	0.584	n.s.	3615.45	132.37	3.66
2	0.001	***	2284.84	236.62	10.36
3	0.012	**	4364.57	518.56	11.88
4	0.928	n.s.	1516.97	262.08	17.28
5	0.769	n.s.	574.64	56.14	9.77

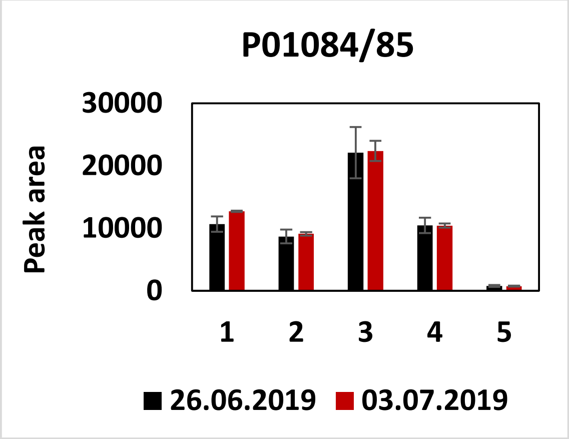

Nr.	t-Test	Significance	Mean	SD	Rel. SD
1	0.016	*	11700.95	1344.17	11.49
2	0.476	n.s.	8906.97	782.24	8.78
3	0.912	n.s.	22274.03	2635.38	11.83
4	0.971	n.s.	10464.57	833.28	7.96
5	0.505	n.s.	779.18	104.08	13.36

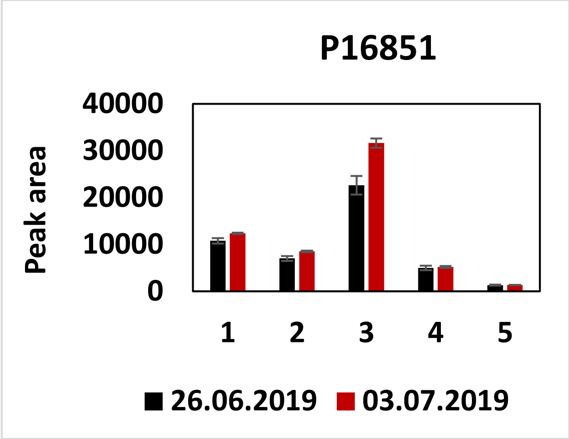

Nr.	t-Test	Significance	Mean	SD	Rel. SD
1	0.002	n.s.	11587.14	922.34	7.96
2	0.002	**	7790.71	883.16	11.34
3	0.000	*	27157.13	5012.38	18.46
4	0.542	n.s.	5118.52	364.37	7.12
5	0.844	n.s.	1356.27	85.82	6.33

1 Julius - Whole meal flour
2 Julius - Centrifuged flour
3 Julius - Passage flour
4 Julius - Grain bran
5 Julius - Sling bran

Figure S7. Relative standard deviation (rel. SD) from the determination of repeatability of the analysis for the grain fractions of the wheat variety Julius (2018). The presented peak areas are related to corresponding 1 µg protein. n.s. = not significant

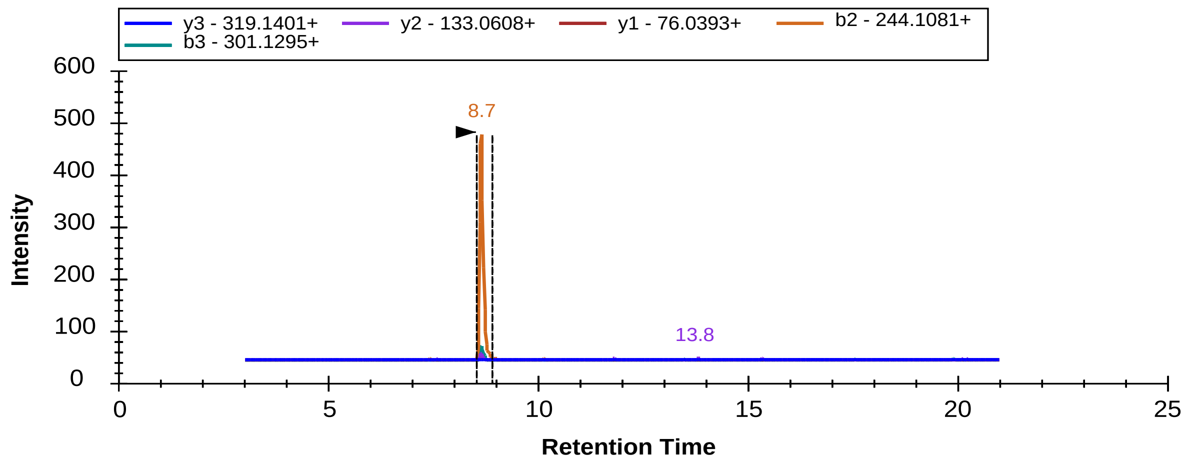

Figure S8. Chromatogram of the peptide GWGG (MS conditions are documented in the Supplementary Table S4)

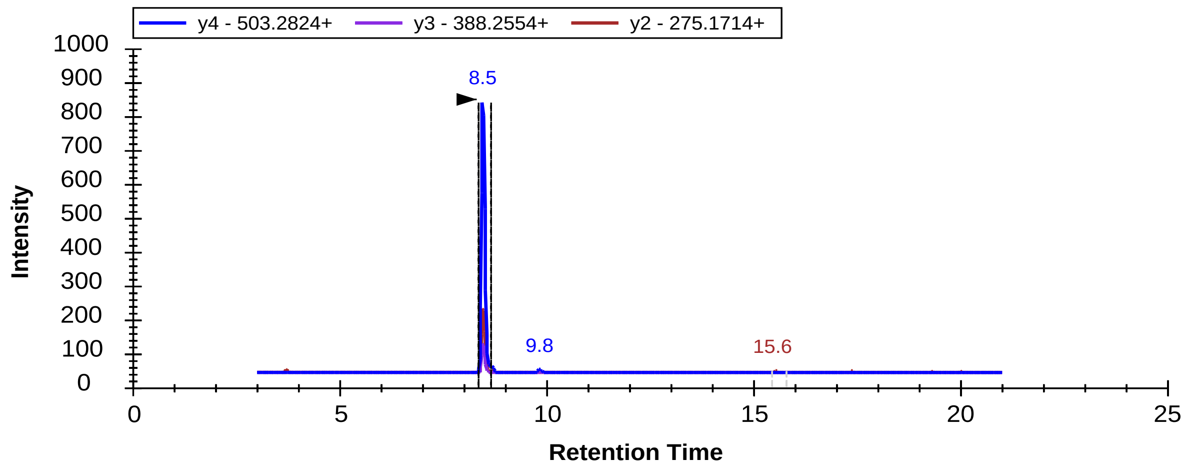

Figure S9. Chromatogram of the peptide K.GLDIQK.V [8, 13] from the protein beta-lactoglobulin added to the samples prior to the digestion (MS conditions are documented in the Supplementary Table S6)

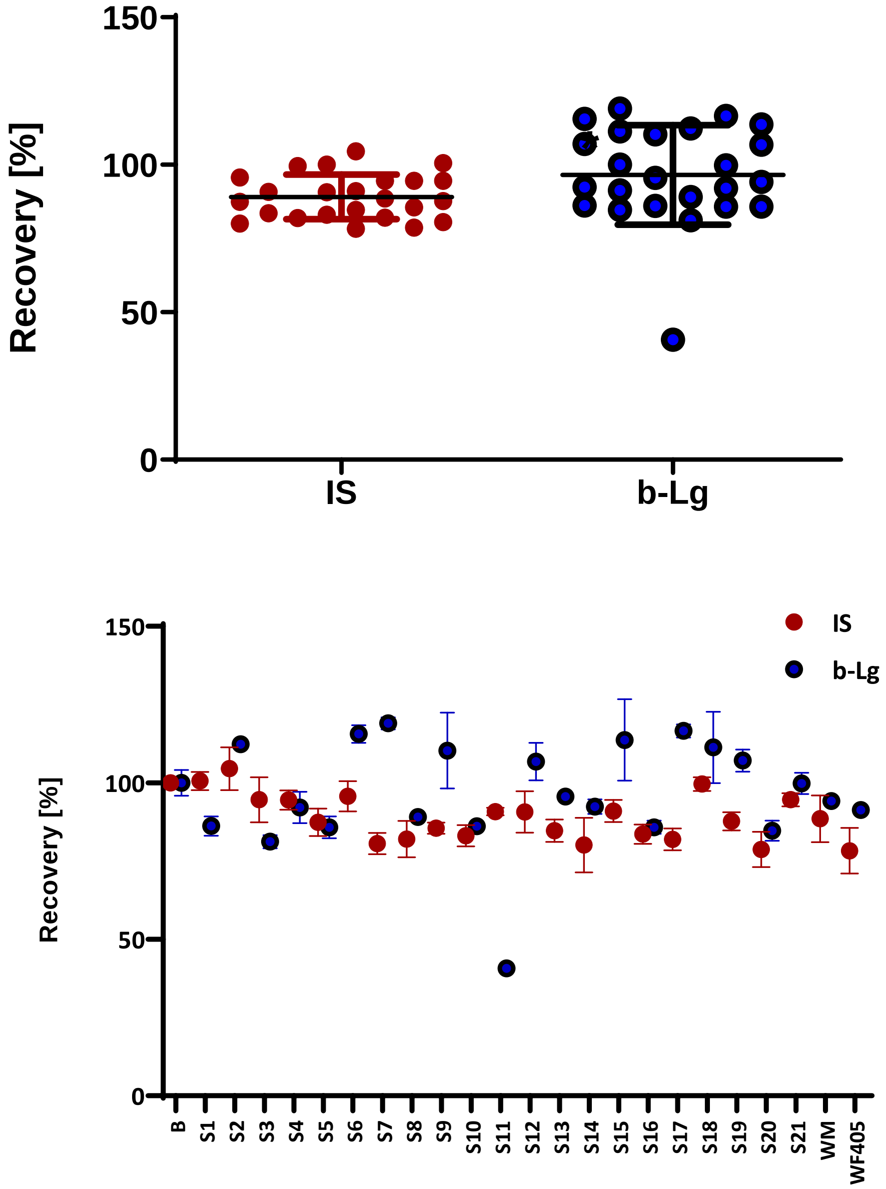

Figure S10. The recovery of IS (internal standard GWGG) and the quantifier peptide for b-Lg used to monitor the digestion (GLDIQK) in all the samples (S1-S21, WM and WF405, allocation given in the materials section) while applying the optimized final extraction method.

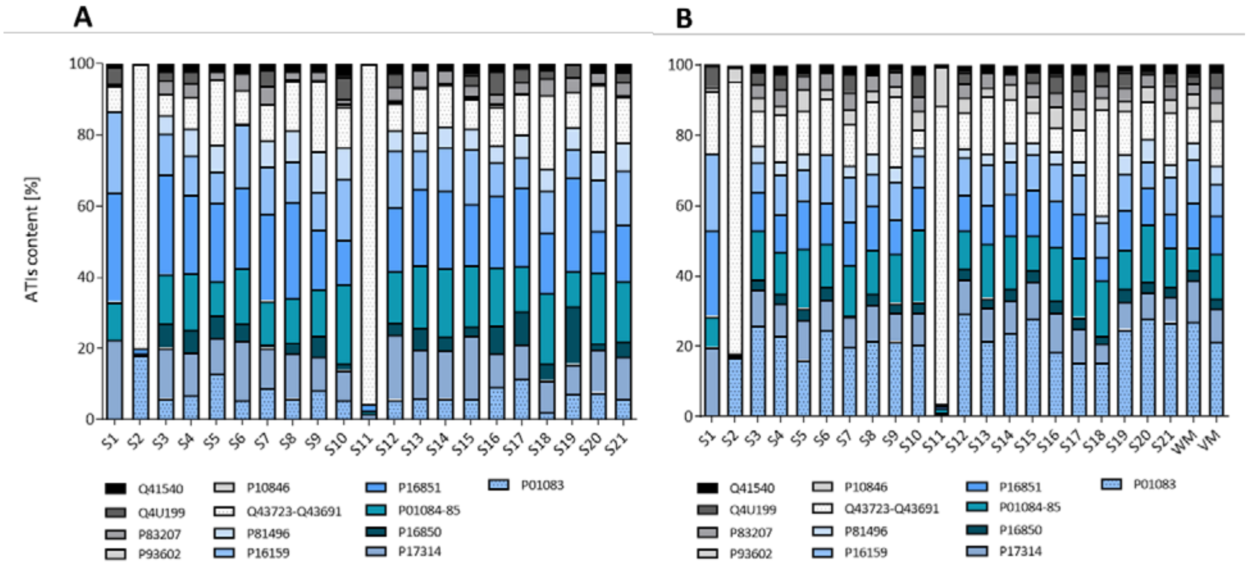

Figure S11. Relative ATI distribution of wheat samples from Turkey carried out using (A) the C/M method and (B) the option 2 of Ambi/urea buffer. S1-S21 represent *Kunduru, Siyazan, Tosunbey, Tosunbey, Esperya, Kayra, Sivas 111/33, Ceyhan-99, Ceyhan-99, Pehlivan, Siyazan, Russian, Esperya, Ceyhan-99, Russian, Pehlivan, Bezostaja, Siyazan, AK-702, Esperya* and *Altay* cultivars, respectively.

Table S1 Preliminary research on amylase/trypsin inhibitors from wheat (UniPtot – as of 15-02-2019) - Data with calculated isoelectric points (pI) and molecular weight of selected ATIs and CM proteins (Wheat; 14 reviewed - Records with information extracted from literature and curator-evaluated computational analysis and 2 Unreviewed - computationally analyzed records that await full manual annotation)

Protein	Uni-Prot access Nr.	pI	Molecular weight (D)	Amino acids	Peptides used in reported studies	Equipment used in the studies considered	Ref.
Alpha-amylase inhibitor **0.19**	P01085 (IAA1_WHEAT)	6.66	13328	1-124	EHGAQEGQAGTGAFPR: Charge 2+; **806.877 →1090.5** (Y11); 806.877 →961.5 (Y10); 806.877 →1346.6 (Y14)	LTQ-Orbitrap; TF;	[2,3]
					LQCNGSQVPEAVLR: Charge 2+; 786.3 →684.3 (Y6); 786.3 →783.5 (Y7); 786.3 → 1330.8 (Y12)	TSQ; TF (CE: 24 V)	[4]
Alpha-amylase inhibitor **0.28**	P01083 (IAA2_WHEAT)	6.19	13326	31-153	SVYQELGVR: Charge 2+; **525.783 →864.5** (Y7); 525.783 →573.3 (Y5); 525.783 →701.4 (Y6)	LTQ-Orbitrap; TF;	[2,3]
					LQCNGSQVPEAVLR: Charge 2+; 786.3 →684.3 (Y6); 786.3 →783.5 (Y7); 786.3 → 1330.8 (Y12)	TSQ; TF (CE: 24/20 V)	[4]
Alpha-amylase inhibitor **WDAI-3**	P10846 (IAA3_WHEAT)	7.57	4797	1-44			
Alpha-amylase inhibitor **0.53**	P01084 (IAA5_WHEAT)	5.23	13185	1-124	LQCNGSQVPEAVLR: Charge 2+; 786.3 →684.3 (Y6); 786.3 →783.5 (Y7); 786.3 → 1330.8 (Y12)	TSQ; TF	[3,4]
Alpha-amylase/trypsin inhibitor **CM1**	P16850 (IAAC1_WHEAT)	6.72	13085	25-145	SDPNSSVLK; Charge 2+; **473.746 →744.4** (Y7); 473.746 →533.3 (Y5); 473.746 →647.4 (Y6)	LTQ-Orbitrap; TF;	[2,3]
Alpha-amylase/trypsin inhibitor **CM2**	P16851 (IAAC2_WHEAT)	6.23	13034	26-145	TSDPNSGVLK; Charge 2+; **509.264 → 829.4** (Y8); 509.264 → 617.4 (Y6); 509.264 → 714.4 (Y7)	LTQ-Orbitrap; TF;	[2,3]
					EYVAQQTCGVGIVGSPVSTEPGNTPR: Charge 3+; 902.2 →641.5 (Y6); 902.2 →958.6 (Y9); 902.2 → 1154.4 (Y11)	TSQ; TF (CE: 18,/24 V)	[4]
					qYVAQQTCGVGIVGSPVSTEPGNTPR: Charge 3+; 896.0 →641.5 (Y6); 896.0 →958.6 (Y9); 896.0 → 1154.4 (Y11)	TSQ; TF (CE: 18/22 V)	[4]
Alpha-amylase/trypsin inhibitor **CM3**	P17314 (IAAC3_WHEAT)	6.66	15832	26-168	YFIALPVPSQPVDPR; Charge 2+; **849.964 →895.5** (Y8); 849.964 →1091.6 (Y10); 849.964 →1204.7 (Y11)	LTQ-Orbitrap; TF;	[2,3,5]
					SGNVGESGLIDLPGCPR; Charge 2+; 864.8 → 585.9 (Y5); 864.8 → 699.3 (Y6); 864.8 → 1185.7 (Y11)	TSQ; TF (CE: 22/30/24 V)	[4]
Alpha-	P16159 (IAC16_WHEAT)	5.02	13437	25-143	QQCCGELANIPQQCR; Charge 2+; 931.8 → 688.6 (Y5); 931.8 → 986.5 (Y8);	TSQ; TF (CE:	[3,4]

amylase/trypsin inhibitor **CM16**					931.8 → 1099.4 (Y9)	26/30/28 V)	
					qQQCCGELANIPQQCR; Charge 2+; 922.9 → 688.6 (Y5); 922.9 → 986.5 (Y8); 922.9 → 1099.4 (Y9)	TSQ; TF (CE: 20/26/24 V)	[4]
Alpha-amylase/trypsin inhibitor **CM17**	P16852 (IAC17_WHEAT)	4.37	3070.31	1-27			[3]
Trypsin/alpha-amylase inhibitor **CMX1/CMX3**	Q43723 (IACX1_WHEAT)	9.10	11400.81	25-121			[3]
Trypsin/alpha-amylase inhibitor **CMX2**	Q43691 (IACX2_WHEAT)	8.93	11458.82	25-121			
Chymotrypsin inhibitor **WCI**	P83207 (ICIW2_WHEAT)	7.42	12935.31	1-119			
Allergen C-C	P81496 (ALCC_WHEAT)	4.94	3136.52	1-27			
PUP88 protein; member of trypsin/a-amylase inhibitors family from cereals	P93602 (P93602_WHEAT)	6.35	14141.28	25-153			
Alpha amylase/trypsin inhibitor	A0A1S6KXP9 (A0A1S6KXP9_WHEAT)	7.66	13060.10	8-124			

Quantification was based on the area of the peak as normalized against the area of the b3 ion of the spiked YGGFL-NH2 peptide. [2]

The quantifier is highlighted in red

TF: Thermo-Fisher Scientific; CE: Collision energy in V

Alpha-amylase/trypsin inhibitor CMX2 is obsolete; deleted from the Databank as of 07.11.2018

Remarks:

HPLC-MS/MS and Columns used in [2]: Tryptic Digestion; LTQ-Orbitrap VELOS mass spectrometer (i.e., a Linear ion Trap Quadrupole mass filter associated with an Orbitrap™ analyzer, Thermo-Fisher Scientific) coupled to a nanoscale liquid-chromatography system; Column - Acclaim® PepMap™ C18 2-µm 100 A, 75-µm i.d. x 15-cm long, Thermo-Fisher Scientific/Dionex)

HPLC-MS and Columns used in [3]: Identification of 10 ATI subtypes (0.28, 0.53, 0.19, CM16, CM17, CM1, CM2, CM3, CMX1, and CMX3), with 0.19 and CM3 representing > 50% of the total ATIs; Peptic / Tryptic Digestion - Waters nanoAcquity System equipped Nanoelectrospray Source on a Waters Synapt G2-S mass spectrometer; Column: C18 HSS-T3 75 µm x 150 mm column

HPLC-MS and Columns used in [5]: Peptic / Chymotryptic / Tryptic Digestion - UPLC/ ESI-MS system (UPLC Acquity Waters with a single quadrupole mass spectrometer Waters Acquity Ultraperformance); Column: ACQUITY UPLC BEH 300 C18 1.7 µm 2.1 x 150 mm (Waters).

HPLC-MS and Columns used in [4]: Predominant ATIs 0.19, 0.28, 0.53, CM2, CM3, and CM16; Tryptic hydrolysis (0.5 mL, enzyme-to-substrate ratio 1:50, 0.04 mol/L urea in 0.1 mol/L Tris-HCl, pH 7.8) was performed for 24 h at 37 °C in the dark; UltiMate 3000 HPLC system (Dionex, Idstein, Germany) coupled to a triple-stage quadrupole mass spectrometer (TSQ Vantage, ThermoFisher Scientific, Bremen, Germany); Column: Aqua-C18 column (50 × 2 mm, 5 µm, 12.5 nm, Phenomenex, Aschaffenburg, Germany

Alignment of the data available for Wheat ATIs for UniProt Database (15-01-2019)

```
P01083/1-153       1 MWMKTVFWGLLVFMLVATTMAVEYGARSHNSGPWSWCNPATGYKVSALTGCRAMVKLQCVGSQV-------------------PEAVLRDCCQQLADINNEWCRCGDLSSMLRSVYQEL-----GVRE 104
P16159/1-143       1 MASKS----NCVLL-LAAVLVSIFAAVAAI--GNEDCTPWMSTLITPLPSCRDYVEQQACRI----------------ETPGSPYLAKQQCCGELANI-PQQCRCQALRYFMGPK----------SRP 94
P17314/1-168       1 MACKS----SCSLLLLAAVLLSVLAAAS----ASGSCVPGVAFRTNLLPHCRDYVLQQTCGTFTPGSKLPEWMTSASIYSPGKPYLAKLYCCQELAEI-SQQCRCEALRYFIALPVPSQPVDPRSGNV 119
P01085/1-124       1 ------------------------------SGPWM-CYPGQAFQVPALPACRPLLRLQCNGSQV-------------------PEAVLRDCCQQLAHI-SEWCRCGALYSMLDSMYKEH-----GAQE 72
P16850/1-145       1 MASKS----SISPLLLATVLVSVFAAATAT---GPYCYAGMGLPINPLEGCREYVAQQTCGISISGSAVST--------EPG--NTPRDRCCKELYDA-SQHCRCEAVRYFIGRR----------SDP 100
P01084/1-124       1 ------------------------------SGPWM-CYPGQAFQVPALPGCRPLLKLQCNGSQV-------------------PEAVLRDCCQQLADI-SEWPRCGALYSMLDSMYKEH-----GVSE 72
P16851/1-145       1 MASKS----SITHLLLAAVLVSVFAAAAAT---GPYCYPGMGLPSNPLEGCREYVAQQTCGVGIVGSPVST--------EPG--NTPRDRCCKELYDA-SQHCRCEAVRYFIGRT----------SDP 100
P10846/1-44        1 ------------------------------SGPWM-CYPGYAFKVPALPGCRPVLLLQCNGSQV-------------------PEAVLRDCCQQ---------------------------------- 44
P16852/1-27        1 ---------------------------------NEDCTPWTSTLIXPLPXCRNYVXXQAC-------------------------------------------------------------------- 27
P83207/1-119       1 -----------------------------T--SIYTCYEGVGLPVDPLQGCHYYVTSQTCGFVPL----------------LPIEVMKDRCCRELAAI-SSNCRCEGLRVFIDRAFPPSQSQ--GGGP 78
Q43723/1-121       1 MAFKH------QLILSTAILLAVLAAASAS--FREQCVPGREITYESLNARREYAVRQTCGYYLS------------------AERQKRRCCDELSKV-PELCWCEVLRILMDRRVT------KEGVV 95
Q43691/1-121       1 MAFKH------QLILSTAILLAVLAAASAS--FREQCVPGREITYESLNARREYAVRQTCGYYLS------------------AERQKRRCCDELSKV-PELCWCEVLRILMDRRVT------KEGVV 95
P81496/1-27        1 -----------------------------S--FREQCVPGREITYECLNACAEYAVRQ---------------------------------------------------------------------- 27
P93602/1-153       1 MASNH-----RRFLLSGAVLLSVLAAVAALESVEDECQPGVAFPHNALATCHTYVIKRVCGR-----------------GPSRPMLVKERCCRELAVV-PDYCRCEALRVLMDGVR------AEEGHV 99
A0A1S6KXP9/1-133   1 -------------------------------------------RTNLLPHCRDYVLQQTCGTFTPGSKLPEWMTSASIYSPGKPYLAKLYCCQELAEI-SQQCRCEALRYFIALPVPSQPVDPRSGNV 84

P01083/1-153     105 G---KEVLPGCRKEVMKLTAAS--VPEVCKVPIPNPSGDRAGVCYGDWAAYPDV- 153
P16159/1-143      95 DQSGLMELPGCPREVQMDFVRILVTPGYCNLTTVHNTPYCLAMEESQWS------ 143
P17314/1-168     120 GESGLIDLPGCPREMQWDFVRLLVAPGQCNLATIHNVRYCPAVEQPLWI------ 168
P01085/1-124      73 GQAGTGAFPRCRREVVKLTAAS--ITAVCRLPIVVDASGDGAYVCKDVAAYPDA- 124
P16850/1-145     101 NSSVLKDLPGCPREPQRDFAKVLVTSGHCNVMTVHNAPYCLGLDI---------- 145
P01084/1-124      73 GQAGTGAFPSCRREVVKLTAAS--ITAVCRLPIVVDASGDGAYVCKDVAAYPDA- 124
P16851/1-145     101 NSGVLKDLPGCPREPQRDFAKVLVTPGHCNVMTVHNTPYCLGLDI---------- 145
P10846/1-44          -------------------------------------------------------
P16852/1-27          -------------------------------------------------------
P83207/1-119      79 P--QPPLAPRCPTEVKRDFARTLALPGQCNLPTIHGGPYCVFP------------ 119
Q43723/1-121      96 KGSLLQDMSRCKK-LTREFIAGIVGRE---------------------------- 121
Q43691/1-121      96 KDSLLQDMSRCKK-LTREFIAGIVGRE---------------------------- 121
P81496/1-27          -------------------------------------------------------
P93602/1-153     100 VEGRLGDRRDCPREAQREFAATLVTAAECNLPTVSGVGSTLGAT-GRWMTIELPK 153
A0A1S6KXP9/1-133  85 GESGLIDLPGCPRQMQWDFVRLLVAPGQCNLATIHNVRYCPAVEQPLWI------ 133
```

Alignment of three ATIs with common peptide according to [4]:

```
P01083/1-153     1 MWMKTVFWGLLVFMLVATTMAVEYGARSHNSGPWSWCNPATGYKVSALTGCRAMVKLQCVGSQVPEAVLRDCCQQLADINNEWCRCGDLSSMLRSVYQELGVREG---KEVLPGCRKEVMKLTAASVP 125
P01085/1-124     1 ------------------------------SGPWM-CYPGQAFQVPALPACRPLLRLQCNGSQVPEAVLRDCCQQLAHI-SEWCRCGALYSMLDSMYKEHGAQEGQAGTGAFPRCRREVVKLTAASIT 96
P01084/1-124     1 ------------------------------SGPWM-CYPGQAFQVPALPGCRPLLKLQCNGSQVPEAVLRDCCQQLADI-SEWPRCGALYSMLDSMYKEHGVSEGQAGTGAFPSCRREVVKLTAASIT 96

P01083/1-153   126 EVCKVPIPNPSGDRAGVCYGDWAAYPDV 153
P01085/1-124    97 AVCRLPIVVDASGDGAYVCKDVAAYPDA 124
P01084/1-124    97 AVCRLPIVVDASGDGAYVCKDVAAYPDA 124
```

Alignment of three ATIs with common peptides CMX1/2/3 and Allergen C-C

```
Q43723/1-121     1 MAFKHQLILSTAILLAVLAAASASFREQCVPGREITYESLNARREYAVRQTCGYYLSAERQKRRCCDELSKVPELCWCEVLRILMDRRVTKEGVVKGSLLQDMSRCKKLTREFIAGIVGRE 121
Q43691/1-121     1 MAFKHQLILSTAILLAVLAAASASFREQCVPGREITYESLNARREYAVRQTCGYYLSAERQKRRCCDELSKVPELCWCEVLRILMDRRVTKEGVVKDSLLQDMSRCKKLTREFIAGIVGRE 121
P81496/1-27      1 -----------------------SFREQCVPGREITYECLNACAEYAVRQ----------------------------------------------------------------------- 27
```

Table S2 Final selection of the known amylase/trypsin inhibitors (UniProt KB, 20.01.2020) analyzed in this work = green; confirmed = *

**Protein**	**UniProt Name**	**pI**	**Molecular weight (D)**	**Length (AS)**
.	P01085	6.66	13328.4	124
α-Amylase Inhibitor **0.28** *	P01083	7.45	16788,1	153
α-Amylase Inhibitor **WDAI-3 ***	P10846	7.57	4793.3	44
α-Amylase Inhibitor **0.53 ***	P01084	5.23	13185.2	124
α-Amylase/Trypsin Inhibitor **CM1 ***	P16850	7.50	15506.5	145
α-Amylase/Trypsin Inhibitor **CM2 ***	P16851	6.86	15449.5	145
α-Amylase/Trypsin Inhibitor **CM3 ***	P17314	7.43	18209.1	168
α-Amylase/Trypsin Inhibitor **CM16 ***	P16159	5.31	15771.6	143
α-Amylase/Trypsin Inhibitor **CM17** (Fragment) *	P16852	4.37	3070.31	27
Trypsin/α-Amylase Inhibitor **CMX1/CMX3 ***	Q43723	9.23	13822.2	121
Trypsin/α-Amylase Inhibitor **CMX2 ***	Q43691	9.08	13880.2	121
Chymotrypsin Inhibitor **WCI ***	P83207	7.42	12935.3	119
**Allergen C-C ***	P81496	4.94	3134.4	27
Trypsin/α-Amylase Inhibitor Protein **PUP88**	P93602	7.56	16580.4	153
dimerer α-Amylase Inhibitor	Q4U199	5.30	14988.2	141
α-Amylase/Trypsin Inhibitor **CM17**	Q41540	5.07	15978.6	143

Table S3 Final selection and assignment of the ATI peptides used for analysis; AA = Amino acid; Qual. = qualifier; Quant. = quantifier. Similarities to other entries in the database is documented by alignment of the sequences in the Excel file supplementary information "selection of peptides"

Protein	Peptide	Assignment	Remarks
P01083	K.VSALTGCR.A	Quant./Qual.	
	K.LTAASVPEVCK.V	Qual.	
	K.VPIPNPSGDR.A	Quant./Qual.	
P17314	R.TNLLPHCR.D	Quant.	A0A1S6KXP9/ Q53YX8 (identical except for one AA)
	R.CEALR.Y	Qual.	
	R.YFIALPVPSQPVDPR.S	Qual.	
	R.SGNVGESGLIDLPGCPR.E	Qual.	
P16850	R.CEAVR.Y	Qual.	
	R.YFIGR.R	Qual	
	R.SDPNSSVLK.D	Quant.	Similar to A0A4P8DL35
	K.DLPGCPR.E	Qual.	
P01084/85	K.LTAASITAVCR.L	Quant.	Similar to A0A077RSX3
	R.LPIVVDASGDGAYVCK.D	Qual.	
	K.DVAAYPDA	Qual.	
P16851	R.CEAVR.Y	Qual.	
	R.YFIGR.T	Qual.	
	R.TSDPNSGVLK.D	Quant.	Similar to A0A4P8DL25
	K.DLPGCPR.E	Qual.	
P16159	R.DYVEQQACR.I	Qual.	Similar to A0A4P8DL87
	R.YFMGPK.S	Quant./Qual.	Similar to A0A4P8DL87/ A0A3B6JQP1/ Q41540
	R.EVQMDFVR.I	Quant./Qual.	Similar to A0A4P8DL87/ Q41450
P81496-Q43723-Q43691	R.EQCVPGR.E	Quant.	
	R.EITYECLNACAEYAVR.Q	Qual.	
	R.EFIAGIVGR.E	Qual.	Similar to A0A4V1DXH0/ Q43691
P93602	R.ELAVVPDYCR.C	Qual.	
	R.CEALR.V	Quant./Qual.	
	R.VLMDGVR.A	Qual.	
	R.WMTIELPK.	Quant./Qual.	
P83207	R.ELAAISSNCR.C	Quant.	
	R.CEGLR.V	Qual.	
	R.VFIDR.A	Qual.	
Q4U199	K.LTAASITAVCK.L	Quant.	
	K.LPIVIDASGDGAYVCK.G	Qual.	
	K.GVAAYPDA	Qual.	
Q41540	R.NYVEEQACR.I	Quant./Qual.	Similar to A0A3B6JQP1
	R.IEMPGPPYLAK.Q	Quant./Qual	Similar to A0A3B6JQP1
	R.YFMGPK.S	Qual.	Similar to A0A4P8DL87/ A0A3B6JQP1/ P16159

Table S4A. Protein content of the CM extracts from German wheat cultivars as determined by the LOWRY assay

Samples	Protein content [mg/ml]
Julius - Whole meal flour	0.834 ± 0.058
Julius - Centrifuged flour	0.777 ± 0.010
Julius - Passage flour	0.451 ± 0.037
Julius - Grain bran	1.199 ± 0.035
Julius - Sling bran	0.806 ± 0.038
Ponticus - Whole meal flour	0.634 ± 0.071
Ponticus - Centrifuged flour	1.104 ± 0.054
Ponticus - Passage flour	0.539 ± 0.010
Ponticus - Grain bran	0.958 ± 0.015
Ponticus - Sling bran	0.936 ± 0.028
Elixier - Whole meal flour	0.926 ± 0.028
Elixier - Centrifuged flour	1.057 ± 0.041
Elixier - Passage flour	0.517 ± 0.053
Elixier - Grain bran	0.595 ± 0.010
Elixier - Sling bran	0.749 ± 0.021
Julius - 2017	0.509 ± 0.005
RGT Reform	0.657 ± 0.030
Findus	0.639 ± 0.022
Nordkap	0.505 ± 0.033
Patras	0.632 ± 0.010
Ponticus 2017	0.620 ± 0.002
Kerubino	0.470 ± 0.006
Tobias	0.523 ± 0.021
Capo	0.378 ± 0.003
Ackermanns Bayernkönig	0.591 ± 0.011

As already mentioned in the introduction, various methods are currently used to extract ATIs. They are mostly based on their solubility in water- and salt-containing buffers [5,6]. The extraction method used in this work combines the good solubility of ATIs in chloroform/methanol mixtures as well as in aqueous buffer systems [1]. The effectiveness of the chosen extraction method can be assessed by means of the two methods used to analyze the proteins in the extracts and a comparison with the literature. The relevant results are summarized in Table 1 and the supplementary information Table S4A. The data given in Table 4A also corresponds to µg/mg flour. The results of the Lowry method and SDS-PAGE show that the extraction of ATIs was basically successful. Protein (Lowry) or bands between 12-16 kDa (SDS-PAGE) could be detected in all samples, in varying degrees. According to general opinion in the literature, ATIs from wheat are found in this molecular weight range. One way to estimate the effectiveness of the CM extraction is to compare the measured protein content of the extracts with the theoretical ATI content of the literature. The prerequisite is that only the desired ATIs have been extracted. The literature shows that ATIs account for approximately 2-4% of the protein content in wheat grain [7]. The theoretical ATI content can therefore be calculated from the protein content of the sample. The total protein content of the different samples was determined by using the Kjeldahl method and is summarized in the Table 1. Overall, the protein content varies between 11-19%, which is roughly in line with the literature value of 10-15% [8]. An average value of 3% was used to calculate the theoretical ATI content. This results in a theoretical ATI content of about 3-6 µg/mg weighed flour. A comparison of the measured protein amount with the

theoretical ATI content shows that the measured values are only in the range of 9-24% of the theoretical content. Thus, the effectiveness of the extraction method is relatively low.

Table S4B. Protein concentration of wheat samples from Turkey. The protein extraction was performed with a chloroform/methanol mixture (C/M method) and with an extraction buffer consisting of ammonium bicarbonate and urea (option 2). The determination of the protein concentration was performed by the Lowry method. In addition, the Kjeldahl method was used to determine the protein content in selected samples, besides the data provided in Table 2.

	**Protein concentration [mg/ml]**			**Protein contentt**
**Sample**	**C/M-Method**		**Option 2**	**[g/100 g]** [A]
S1	0.27 ± 0.00	*	6.10 ± 0.20	
S2	0.47 ± 0.01	*	7.07 ± 0.11	12.1
S3	0.46 ± 0.02	*	6.45 ± 0.05	
S4	0.39 ± 0.02	*	5.37 ± 0.16	
S5	0.53 ± 0.04	*	7.11 ± 0.11	
S6	0.47 ± 0.02	*	5.66 ± 0.36	
S7	0.46 ± 0.01	*	7.13 ± 0.21	
S8	0.60 ± 0.09	*	6.65 ± 0.11	
S9	0.75 ± 0.03	*	6.89 ± 0.09	
S10	1.23 ± 0.03	*	6.63 ± 0.17	
S11	0.38 ± 0.00	*	8.49 ± 0.27	15.6
S12	0.63 ± 0.09	*	7.13 ± 0.16	
S13	0.44 ± 0.03	*	6.53 ± 0.22	
S14	0.41 ± 0.01	*	6.59 ± 0.20	
S15	0.60 ± 0.00	*	6.77 ± 0.24	13.1
S16	0.52 ± 0.00	*	7.82 ± 0.15	
S17	0.61 ± 0.03	*	5.85 ± 0.11	
S18	0.37 ± 0.03	*	6.19 ± 0.20	
S19	0.27 ± 0.03	*	5.99 ± 0.32	
S20	0.66 ± 0.06	*	5.87 ± 0.12	13.9
S21	0.54 ± 0.02	*	4.76 ± 0.13	

[A] Protein determination according to Kjeldahl Method; S1-S21: Wheat samples (see Table2); * marks significant difference between the extraction methods ($p < 0.05$).

Table S5. Percentage of fractions of whole grain for the wheat varieties Julius 2018. Ponticus 2018 and Elixer (the data is based on the grinding protocol applied and documented in supplementary Figure S1)

**Grain fractions**	**Julius [%]**	**Ponticus [%]**	**Elixer [%]**
Whole meal flour	100	100	100
Centrifuged flour	8	8	8
Passaged flour	63	64	67
Grain bran	25	26	24
Sling bran	20	21	19

Table S6 The optimized conditions for the multiple reaction monitoring (MRM) for the final HPLC-MS/MS method; Q1 = Precursor mass; Q3 = Transition mass; CE = Collision energy

Protein	Sequence	Fragment	Q1-Mass	Q3-Mass	CE [eV]	Retention time [min]
P01083	K.VSALTGCR.A	A[y6]	432.2^{++}	677.3^{+}	14.4	8.0
		L[y5]		606.3^{+}	14.4	
		T[y4]		493.2^{+}	14.4	
P01083	K.VPIPNPSGDR.A	P[y9]	526.3^{++}	952.5^{+}	23.3	9.1
		P[y7]		742.3^{+}	23.3	
		P[y5]		531.3^{+}	26.3	
P17314	R.TNLLPHCR.D	L[y6]	505.8^{++}	795.4^{+}	19.7	8.6
		P[y4]		569.3^{+}	19.7	
		L[b3]		329.2^{+}	19.7	
P16850	R.SDPNSSVLK.D	P[y7]	473.7^{++}	744.4^{+}	15.7	8.1
		S[y5]		533.3^{+}	21.7	
P01084/85	K.LTAASITAVCR.L	T[y10]	581.8^{++}	1049.5^{+}	25.0	10.2
		S[y9]		806.4^{+}	22.0	
		T[y5]		606.3^{+}	25.0	
P15851	R.TSDPNSGVLK.D	P[y7]	509.3^{++}	714.4^{+}	19.8	8.1
		S[y5]		503.3^{+}	25.8	
		P[y7]		357.7^{++}	19.8	
P16159	R.YFMGPK.S	F[y5]	371.7^{++}	579.3^{+}	12.5	9.7
		M[y4]		432.2^{+}	12.5	
		G[y3]		301.2^{+}	12.5	
		P[y2]		244.2^{+}	12.5	
P16159	R.EVQMDFVR.I	Q[y6]	512.3^{++}	795.4^{+}	16.9	10.9
		M[y5]		667.3^{+}	16.9	
		D[y4]		536.3^{+}	13.9	
		F[y3]		421.3^{+}	25.9	
P81496-Q43723-Q43691	R.EQ<u>C</u>VPGR.E	C[y5]	423.2^{++}	588.3^{+}	14.0	6.85
		V[y4]		428.3^{+}	14.0	
		P[y3]		329.2^{+}	14.0	
P93602	R.<u>C</u>EALR.V	E[y4]	324.7^{++}	488.3^{+}	14.1	6.95
		A[y3]		359.2^{+}	11.1	
		L[b4]		474.2^{+}	11.1	
P83207	R.ELAAISSN<u>C</u>R.C	A[y7]	560.8^{++}	807.4^{+}	18.4	8.9
		I[y6]		736.3^{+}	21.4	
		S[y5]		623.3^{+}	21.4	
		S[y4]		536.2^{+}	18.4	
Q4U199	K.LTAASITAV<u>C</u>K.L	T[y10]	567.8^{++}	1021.5^{+}	25.0	10.0
		S[y7]		778.4^{+}	22.0	
		T[y5]		578.3	25.0	
Q41540	R.NYVEEQACR.I	Y[y8]	608.3^{++}	1054.5^{+}	25.1	8.0
		V[y7]		891.4^{+}	19.1	
		E[y6]		792.3^{+}	19.1	
		E[y5]		663.3^{+}	19.1	
Q41540	R.IEMPGPPYLAK.Q	M[y9]	608.3^{++}	973.5^{+}	13.9	11.8
		P[y8]		842.5^{+}	28.9	
		G[y6]		745.4^{+}	19.9	
		P[y6]		688.4^{+}	13.9	

Table S6 continued

Protein	Sequence	Fragment	Q1-Mass	Q3-Mass	CE [eV]	Retention time [min]
P02754	K.GLDIQK.V	D[Y4]	337.2^{++}	503.3^{+}	8.5	8.5
(β-lactoglobulin)		I[y3]		388.3^{+}	11.5	
		Q[y2]		275.2^{+}	14.5	
IS	GWGG	W[y3]	376.2^{+}	319.1^{+}	9.7	8.7
		G[y2]		133.1^{+}	9.7	
		G[y1]		76.0^{+}	15.7	
		W[b2]		244.1^{+}	9.7	
		G[b3]		301.1^{+}	9.7	

Table S7. Summary of recovery tests for the CM method and the optimized final extraction method (Section 2.2.1/2.2.7 in the manuscript).

Sample*	Quantification peptide applied / Method	Recovery [%]
IS in buffered blank	GWGG / CM – Section 2.2.1	100
IS in Julius 2018. whole meal flour		89
IS in Julius 2018. centrifuged flour		80
IS in Julius 2018. passaged flour		78
IS in Julius 2018. grain bran		87
IS in Julius 2018. sling bran		84
IS in buffered blank	GWGG / Second option – Section 2.2.7.1	100
IS with sample S15		91
IS with sample S20		79
IS with sample WF405		78
IS with sample WM		89
b-Lg in buffered blank	GLDIQK / CM – Section 2.2.1	100
b-Lg with sample S15		92
b-Lg with sample S20		105
b-Lg with sample WF405		97
b-Lg with sample WM		97
b-Lg in buffered blank	GLDIQK / Second option – Section 2.2.7.1	100
b-Lg with sample S15		113
b-Lg with sample S20		79
b-Lg with sample WF405		83
b-Lg with sample WM		85

*** For the sample allocation please see materials section.**

References

1. Sagu, S.T.; Huschek, G.; Bonick, J.; Homann, T.; Rawel, H.M. A New Approach of Extraction of alpha-Amylase/trypsin Inhibitors from Wheat (Triticum aestivum L.), Based on Optimization Using Plackett-Burman and Box-Behnken Designs. *Molecules* **2019**, *24*, doi:ARTN 3589; 10.3390/molecules24193589.

2. Rogniaux, H.; Pavlovic, M.; Lupi, R.; Lollier, V.; Joint, M.; Mameri, H.; Denery, S.; Larre, C. Allergen relative abundance in several wheat varieties as revealed via a targeted quantitative approach using mass spectrometry. *Proteomics* **2015**, 10.1002/pmic.201400416, doi:10.1002/pmic.201400416.

3. Zevallos, V.F.; Raker, V.; Tenzer, S.; Jimenez-Calvente, C.; Ashfaq-Khan, M.; Russel, N.; Pickert, G.; Schild, H.; Steinbrink, K.; Schuppan, D. Nutritional Wheat Amylase-Trypsin Inhibitors Promote Intestinal Inflammation via Activation of Myeloid Cells. *Gastroenterology* **2017**, *152*, 1100-1113 e1112, doi:10.1053/j.gastro.2016.12.006.

4. Geisslitz, S.; Ludwig, C.; Scherf, K.A.; Koehler, P. Targeted LC-MS/MS Reveals Similar Contents of alpha-Amylase/Trypsin-Inhibitors as Putative Triggers of Nonceliac Gluten Sensitivity in All Wheat Species except Einkorn. *Journal of agricultural and food chemistry* **2018**, *66*, 12395-12403, doi:10.1021/acs.jafc.8b04411.

5. Prandi, B.; Faccini, A.; Tedeschi, T.; Galaverna, G.; Sforza, S. LC/MS analysis of proteolytic peptides in wheat extracts for determining the content of the allergen amylase/trypsin inhibitor CM3: influence of growing area and variety. *Food chemistry* **2013**, *140*, 141-146, doi:10.1016/j.foodchem.2013.02.039.

6. Feng, G.H.; Richardson, M.; Chen, M.S.; Kramer, K.J.; Morgan, T.D.; Reeck, G.R. alpha-Amylase inhibitors from wheat: amino acid sequences and patterns of inhibition of insect and human alpha-amylases. *Insect biochemistry and molecular biology* **1996**, *26*, 419-426.

7. Dupont, F.M.; Vensel, W.H.; Tanaka, C.K.; Hurkman, W.J.; Altenbach, S.B. Deciphering the complexities of the wheat flour proteome using quantitative two-dimensional electrophoresis, three proteases and tandem mass spectrometry. *Proteome science* **2011**, *9*, 10, doi:10.1186/1477-5956-9-10.

8. Tatham, A.S.; Shewry, P.R. Allergens to wheat and related cereals. *Clinical and experimental allergy : journal of the British Society for Allergy and Clinical Immunology* **2008**, *38*, 1712-1726, doi:10.1111/j.1365-2222.2008.03101.x.
